# A potential peptide derived from cytokine receptors can bind proinflammatory cytokines as a therapeutic strategy for anti-inflammation

**DOI:** 10.1038/s41598-018-36492-z

**Published:** 2019-02-19

**Authors:** Shinn-Jong Jiang, Pei-I Tsai, Shih-Yi Peng, Chun-Chun Chang, Yi Chung, Hao-Hsiang Tsao, Hsin-Ting Huang, San-Yuan Chen, Hao-Jen Hsu

**Affiliations:** 1 Department of Biochemistry, School of Medicine, Tzu Chi University, Hualien, 97004 Taiwan; 2 Department of Materials Science and Engineering, National Chiao-Tung University, Hsinchu, 30010 Taiwan; 3 Institute of Medical Sciences, Tzu Chi University, Hualien, 97004 Taiwan; 4 Department of Laboratory Medicine, Tzu Chi Medical Center, Hualien, 97004 Taiwan; 5 Department of Life Sciences, Tzu Chi University, Hualien, 97004 Taiwan

## Abstract

Chronic inflammation is a pivotal event in the pathogenesis of cardiovascular diseases, including atherosclerosis, restenosis, and coronary artery disease. The efficacy of current treatment or preventive strategies for such inflammation is still inadequate. Thus, new anti-inflammatory strategies are needed. In this study, based on molecular docking and structural analysis, a potential peptide KCF18 with amphiphilic properties (positively charged and hydrophobic residues) derived from the receptors of proinflammatory cytokines was designed to inhibit cytokine-induced inflammatory response. Simulations suggested that KCF18 could bind to cytokines simultaneously, and electrostatic interactions were dominant. Surface plasmon resonance detection showed that KCF18 bound to both tumor necrosis factor-α (TNF-α) and interleukin-6, which is consistent with MM/PBSA binding free energy calculations. The cell experiments showed that KCF18 significantly reduced the binding of proinflammatory cytokines to their cognate receptors, suppressed TNF-α mRNA expression and monocyte binding and transmigration, and alleviated the infiltration of white blood cells in a peritonitis mouse model. The designed peptide KCF18 could remarkably diminish the risk of vascular inflammation by decreasing plasma cytokines release and by directly acting on the vascular endothelium. This study demonstrated that a combination of structure-based *in silico* design calculations, together with experimental measurements can be used to develop potential anti-inflammatory agents.

## Introduction

Chronic inflammation is a risk factor for atherosclerosis, restenosis, and arthritis^1–3^. In the pathogenesis of atherogenesis processing, the important initiating step is thought to be the injury of endothelium^4,5^. The adhesion of monocytes to activated endothelial cells coupled with transendothelial migration is indispensable consequence of the inflammatory response in the vasculature, and this inflammatory response occurs continuously throughout the atherogenic course. The inflammatory reaction is coordinated by interplay between leukocytes and endothelial cells and is closely associated with endothelial dysfunction^6^. Leukocyte recruitment to vascular endothelium relies to the interplays of endothelial cell surface proteins E- and P-selectins with their ligands expressed on leukocytes. Vascular cell adhesion molecule-1 (VCAM-1) and intracellular adhesion molecule-1 (ICAM-1) are most conspicuously participated in this course^7–9^. The activation of leukocytes is a complex process involving the release of several soluble proinflammatory cytokines, such as tumor necrosis factor-α (TNF-α), interleukin-6 (IL-6), and IL-1β. These cytokines are important regulators of the inflammatory reaction in the vessel wall. These cytokines also play a critical role in preserving host integrity, and they facilitate white blood cell recruitment to remove the components of invading pathogens to prevent the development of infection^10,11^. Most proinflammatory cytokines are primarily produced in response to infection or disease and contribute to the immune response, inflammation, and endothelial activation^12^. However, dysfunction of cytokines productions may lead to several clinical diseases as mentioned previously. These cytokines can increase endothelial permeability and vasodilation and can disrupt the procoagulant–anticoagulant balance^13,14^. The deregulation of these cytokines may cause direct and indirect host injury. Clinical studies^15–17^ have demonstrated that TNF-α and IL-1β blood levels are significantly elevated in patients with endotoxemia, and that the IL-6 level is increased during infectious episodes^17^. In addition to increasing the expression of several genes, the fundamental property of TNF-α is that it induces its own gene transcription^18^. TNF-α has been shown to upregulate IL-1β and IL-6 release^19^. Moreover, IL-1β has been shown to be a potent inducer of IL-6 secretion^20^.

TNF-α is a major cytokine with a molecular weight of 17.0 kDa; it is mainly secreted and produced by macrophages, lymphocytes, mast cells, monocytes, and fibroblasts after bacterial infection^21,22^. Experiments using anti-TNF-α antibodies indicated that inhibiting TNF-α in bacterial or endotoxin-induced shock models can lead to a significant decrease in the levels of other cytokines^23,24^. The structure of TNF-α was resolved in 1989 (PDB code: 1TNF)^25^. This protein is a β-sandwich composed of 10 antiparallel β sheets. It can activate two cognate receptors: TNF receptor 1 and 2 (TNFR1 and TNFR2, respectively)^26–29^. IL-1β, which has a molecular weight of 17.5 kDa, is mainly produced by macrophages, and exerts a remarkable array of biological effects^30^. In animal models, it induces the upregulation of adhesion molecules on both leukocytes and endothelial cells and induces a shock-like state^31^. IL-1β is involved in various cellular activities, such as cell differentiation, proliferation, and apoptosis. Deregulation of the production of IL-1β may cause numerous autoinflammatory syndromes. IL-1β can bind to its type I IL-1 receptor (IL-1R), which is an early step in IL-1 signal transduction^30^. The structure of the IL-1β–IL-1R complex was resolved in 1997 (PDB code: 1ITB)^32^. IL-6, a 20-kDa protein, is also secreted by monocytes, macrophages, endothelial cells, and fibroblasts for stimulating the immune response^33,34^. Moreover, the deregulation of IL-6 production has been implicated in a wide range of autoimmune diseases, including rheumatoid arthritis, diabetes, depression, and multiple myeloma^35^. The nuclear magnetic resonance (NMR) structure of IL-6 was resolved in 1997 (PDB code: 2IL6)^36^. IL-6 has been identified to interact with the ligand-binding chain IL-6Rα (CD126) and the signal transduction component glycoprotein 130 (gp130)^37–40^.

Although therapies have been applied to improve the clinical outcome of patients with severe inflammation through the removal of inflammatory mediators, most approaches have not provided any sustainable benefits for mortality^41–43^. Therefore, new anti-inflammatory strategies are needed. Over the last decade, peptides have been therapeutically utilized as drugs or antagonists in diverse fields such as neurology, endocrinology, and hematology^44^. Peptides act by binding to specific ligands or cell surface receptors, and bioinformatics and structure databases have been used to design new therapeutic peptides that have higher affinity than native proteins for triggering or inhibiting the signal transduction cascade^44^. Peptide drugs have the strengths of predictable metabolism, bioavailability, high efficacy, safety, and tolerability^44–46^. The advantages of peptide drugs over antibodies include specificity, lower cost, lower cytotoxicity, and immune response induction. Therefore, the development of new effective anti-inflammatory or anti-cancer therapies by using peptide-based antagonists has strong potential in the future. Our previous study proposed a multistep model of IL-8 binding to the chemokine receptor CXCR1^47^ and truncated a polypeptide derived from IL-8 to inhibit the binding of IL-8 to CXCR1 based on molecular docking and molecular dynamics simulations; the polypeptide was validated by *in vitro* SPR detection and cellular assays^48^. The purpose of this study is to develop the antagonist peptide to interfere the binding of cytokines and cognate receptors for decreasing the severity of inflammation as therapeutic treatment. In this study, the potential peptide KCF18 was designed based on structural-based molecular docking and MM/PBSA binding free energy calculations and was then synthesized and verified using *in vitro* surface plasmon resonance (SPR) measurements. Moreover, we demonstrated that KCF18 decreased the binding of proinflammatory cytokines to their cognate receptors, suppressed TNF-α mRNA expression and monocyte binding and transmigration, and alleviated the infiltration of white blood cells in a peritonitis model. Our results suggested that the designed peptide KCF18 effectively diminishes the risk of vascular inflammation by decreasing plasma cytokine release and by directly acting on the vascular endothelium.

## Results

### Determining binding sites of cytokine–cognate receptor complexes

Three proinflammatory cytokines, namely TNF-α, IL-1β, and IL-6, which play a critical role in the inflammatory response, were selected to analyze the binding sites of cytokine–cognate receptor complexes. To design the preferable peptides for binding these three cytokines, the characteristics of each cytokine–receptor complex was first investigated. Subsequently, several amino acids from each receptor on the cytokine–receptor binding interface were selected as the potential peptides to compose a new peptide for inhibiting inflammation. In the absence of the structure of the TNF-α–TNFR1 complex, molecular docking was performed for the binding of TNF-α to the ectodomain of TNFR1. The binding site of the receptor TNFR1 was determined to contain 18 residues, named SEM18 (^63^SENHLRHCLSCSKCRKEM^80^) that imparted more positive charges at one end (Supporting Figure S1A). The binding site of TNF-α was more negatively charged, indicating that electrostatic interactions are crucial for the binding of TNF-α to the receptor TNFR1. The structure of the ectodomain of IL-1R complexed with IL-1β was resolved in 1997^49^ (PDB code: 1ITB), which showed that IL-1β is bound to IL-1R at several sites. The binding site of IL-1R was determined to contain more positively charged residues (^108^QAIFKQKLPVAGD^120^) than other sites, and the charge characteristics of IL-1R were similar to those of TNFR1 (Supporting Figure S1B). In the absence of the structure of the N-terminus of IL-6, the structure was homology modeled from the Phyre2 web server to form a new IL-6 structure with N-terminus, which was equilibrated for 50-ns MD simulations. According to the docking of IL-6 to the ectodomain of the IL-6 receptor, a peptide containing neutral hydrophilic and hydrophobic residues (^162^VDYSTVYFVN^171^) was determined to compose the binding site (Supporting Figure S1C).

### Interactions between cytokines and composite peptide

**Figure 1:**
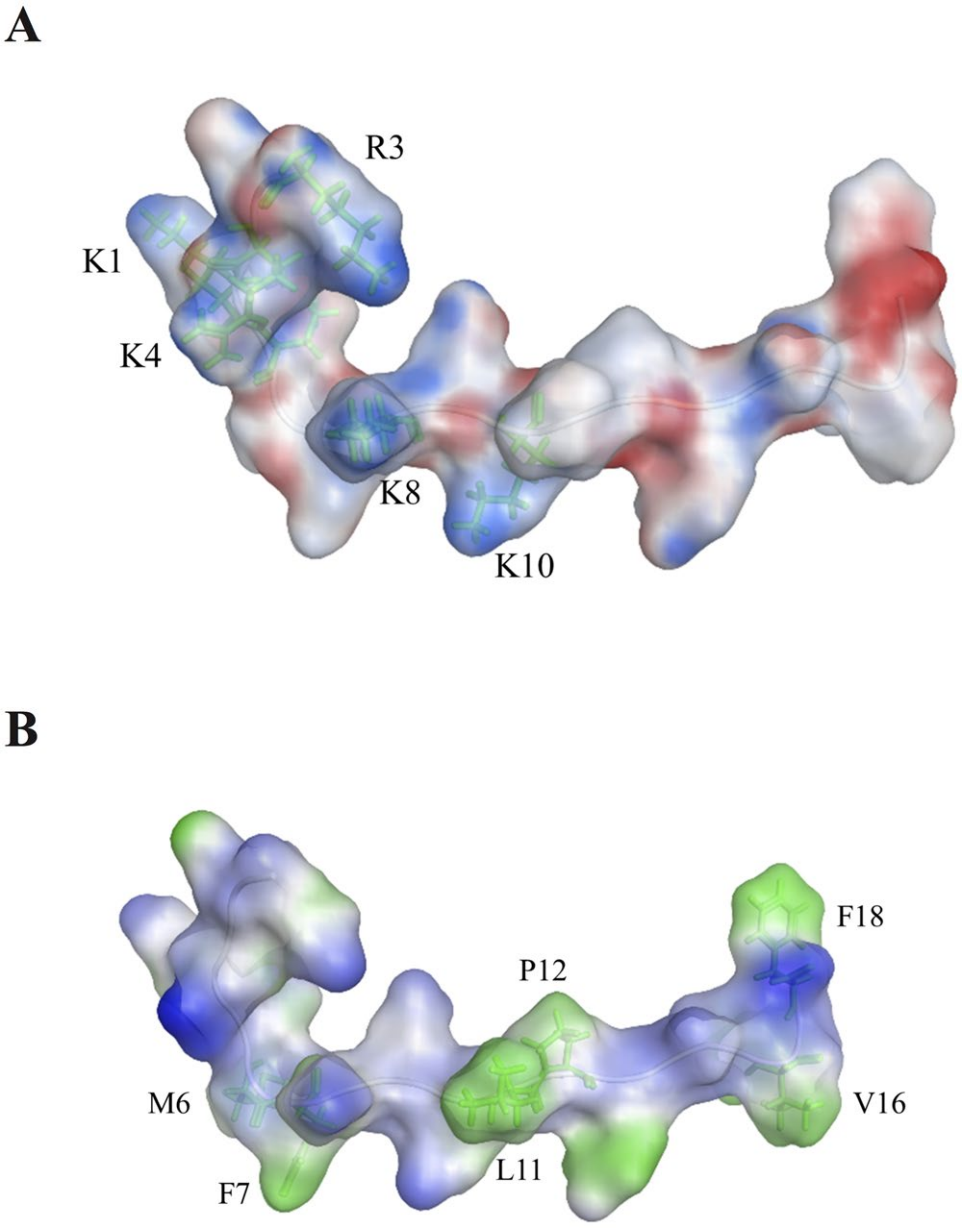
Surface property analysis of the designed peptide KCF18. (**A**) Surface charge distribution of KCF18 was calculated using the Poisson–Boltzmann equation. Blue color corresponds to positive and red color to negative electrostatic potential. KCF18 is more positively charged at one end. (**B**) Surface lipophilicity distribution of KCF18, in which blue color represents hydrophilic residues, whereas green color represents hydrophobic residues. It seems that more hydrophobic residues are distributed at the far end.

**Figure 2:**
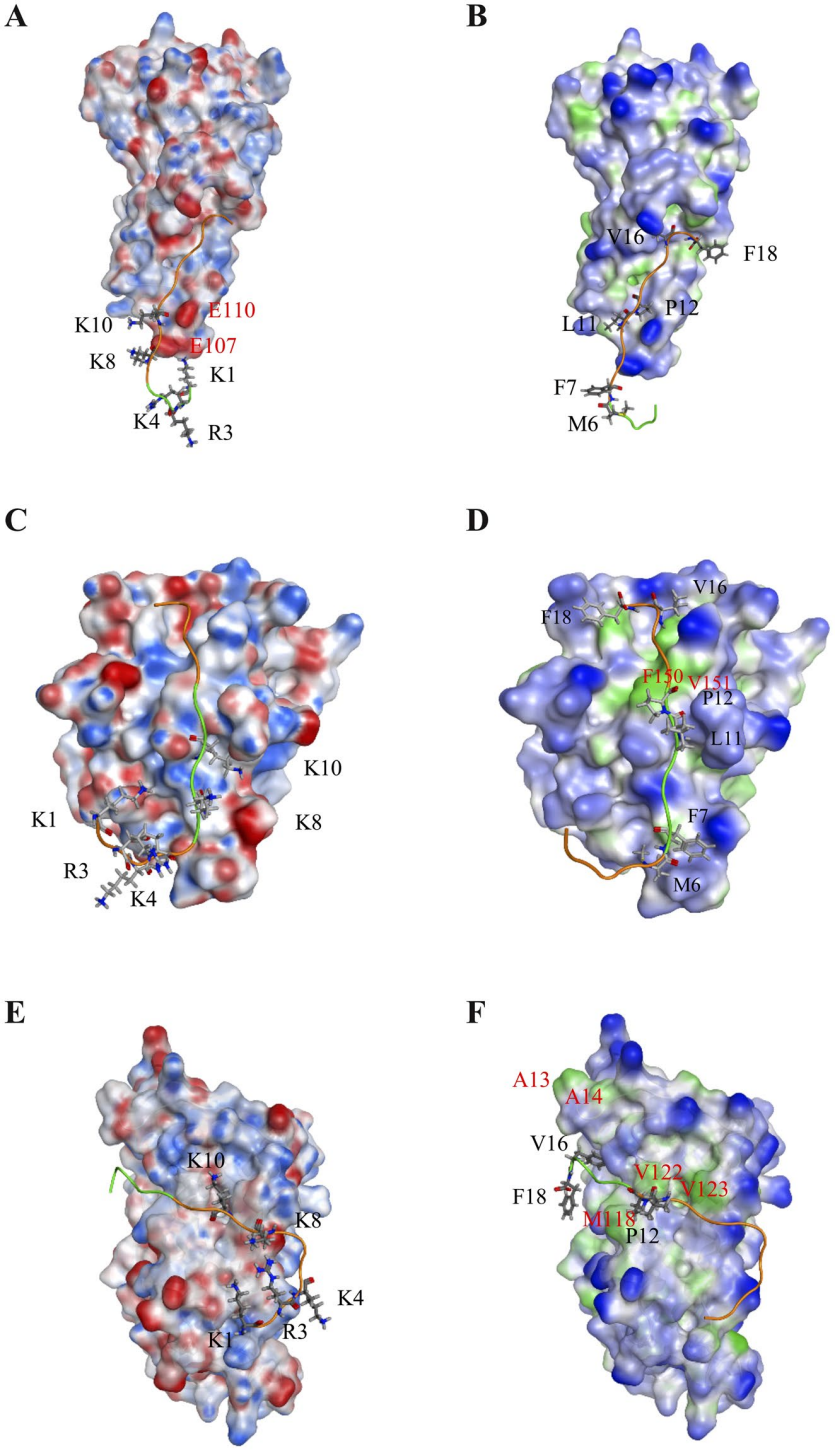
Surface property analysis of preferable binding poses for KCF18 to proinflammatory cytokines. KCF18 is represented as an orange-colored and green-colored loop structure. Residues around the binding interface are labeled and are shown as sticks. Surface charge distribution of KCF18 binding to cytokines was calculated using the Poisson–Boltzmann equation. Blue color corresponds to positive and red color to negative electrostatic potential. (**A**) KCF18–TNF-α, (**C**) KCF18–IL-1β, and (**E**) KCF18–IL-6 complexes. Surface lipophilicity distribution of KCF18 binding to cytokines, in which blue color represents hydrophilic residues, whereas green color represents hydrophobic residues. (**B**) KCF18–TNF-α, (**D**) KCF18–IL-1β, and (**F**) KCF18–IL-6 complexes.

Based on the determined binding sites of the respective cytokine–cognate receptor complex, a new composite peptide (^1^KCRKEMFKQKLPYSTVYF^18^, called KCF18), which contained five positively charged residues and six hydrophobic residues, was designed to bind to the three cytokines simultaneously. Analysis of the surface charge and lipophilicity distributions of the composite peptide revealed that more positively charged residues were present at one end, and more hydrophobic residues were distributed at the other end. Thus, the peptide exhibited amphiphilic properties (Fig. 1). The composite peptide KCF18 was redocked to the three cytokines, showing that the predicted binding site of KCF18 for TNF-α was near the original binding site of the TNF-α–TNFR1 complex. In the predicted binding mode, the positively charged residues of KCF18 (K1, R3, K4, K8, and K10) formed strong electrostatic interactions with the negatively charged residues of TNF-α (E104, E107, and E110) (Fig. 2A). The neutral hydrophobic residues of KCF18 also bound to the hydrophobic residues of TNF-α (Fig. 2B). In the KCF18–IL-1β complex, the redocked binding site was slightly different from the original binding site of the IL-1β–IL-1R complex. In the redocked binding site, the positively charged residues of KCF18 (K1, R3, K4, K8, and K10) were bound to the negatively charged residues of IL-1R (Fig. 2C). The hydrophobic residues of KCF18 (L11 and P12) formed hydrophobic interactions with the hydrophobic residues of IL-1β (F150 and V151) (Fig. 2D). In the KCF18–IL-6 complex, the redocked binding site was near the original binding site of the IL-6–IL-6 receptor complex. The positively charged residues of KCF18 (K1, R3, and K4) were bound to the negatively charged residues of IL-6, and its hydrophobic residues (L11, P12, V16, and F18) also interacted with the hydrophobic residues of IL-6 (A13, A14, M118, V122, and L123) (Fig. 2E,F).

### Binding free energy calculations for the binding of composite peptide to cytokines

To obtain more insights on the molecular docking of the composite peptide to the three cytokines and to determine the dominant interactions between the KCF18 peptide and the cytokines, MM/PBSA binding free energy calculations for the binding of KCF18 to the cytokines were performed, as shown in Supporting Figure S2A. To reduce errors due to limited sampling in the MD simulations, the various complex systems were repeated two times with different initial velocities to perform the first 200 ns MD simulations, indicating that the root-mean-square deviation (RMSD) values of the complex backbone atoms of repeated systems were similar and fluctuated to reach a plateau after 100 ns MD simulations (Supporting Figure S2B). The KCF18 peptide RMSD profiles indicated that the peptide may adopt a stable conformation in all systems after 130 ns MD simulations (Supporting Figure S2C). The binding free energy (Δ*G*_*bind*_) for the binding of the composite peptide KCF18 to the cytokine TNF-α (−583.6 ± 3.6 kJ/mol). The binding free energies for the KCF18–IL-1β and KCF18–IL-6 complexes were −394.9 ± 3.8 and −526.6 ± 5.3 kJ/mol, respectively. The computed binding differences are much larger than the corresponding experimental relative affinities, but indicate that the two cytokines had a higher binding affinity with KCF18. When KCF18 was bound to these cytokines, electrostatic interactions gave the dominant contribution to the computed MM/PBSA binding affinities whereas the contribution due to VDW interactions was minor (Supporting Figure S2A). Moreover, a truncated peptide SEM18 truncated from the binding site of the receptor TNFR1 was also redocked to cytokine TNF-α to calculate MM/PBSA binding free energy (−144.60 ± 4.8 kJ/mol), which is a much higher energy than that of KCF18 to TNF-α complex. This finding implied that the designed peptide KCF18 theoretically has much better binding affinity than the truncated peptide SEM18.

### SPR measurements for the association between composite peptide and cytokines

**Figure 3:**
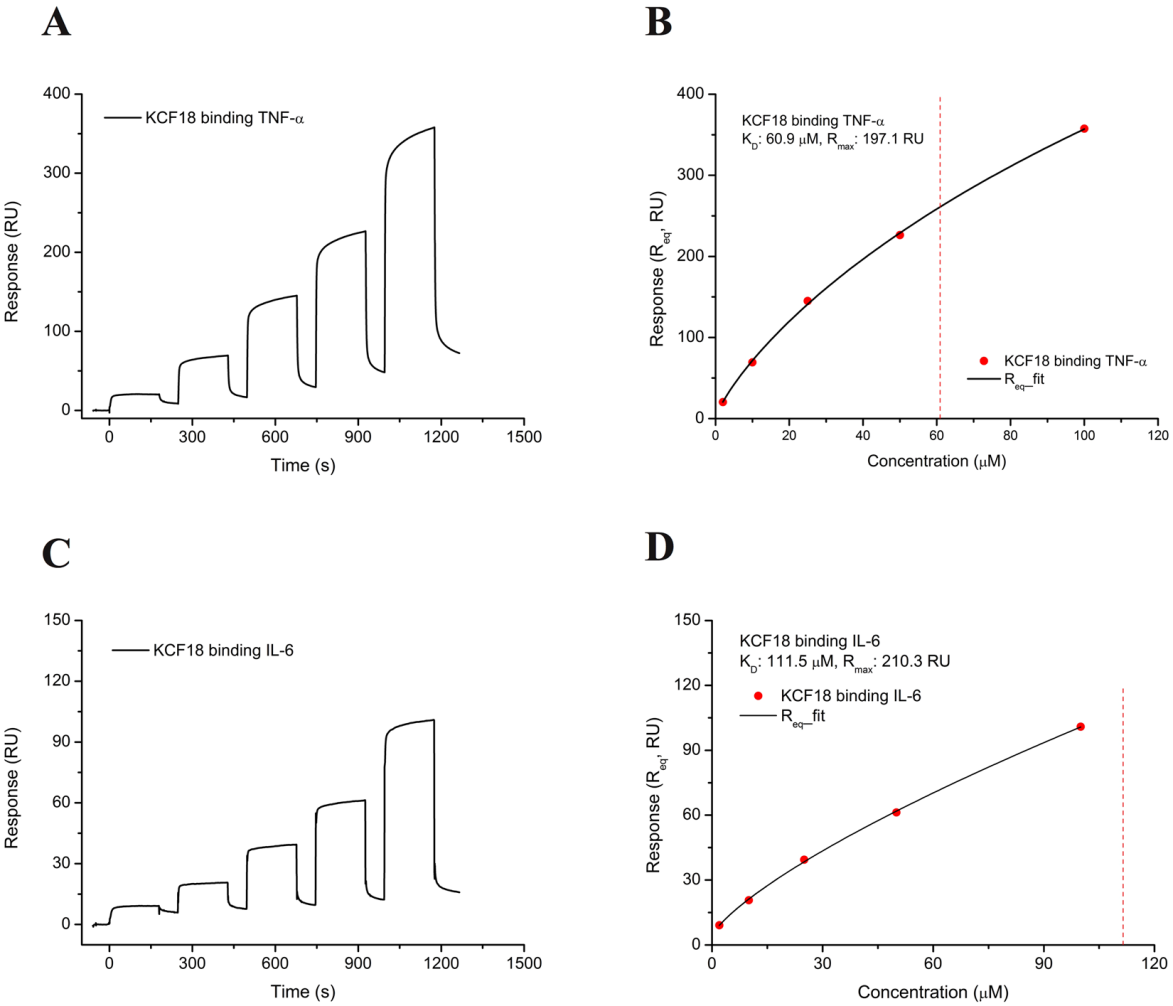
SPR analysis for KCF18 binding to proinflammatory cytokines. (**A**) KCF18 was injected over TNF-α immobilized on the CM5 sensor chip. As the concentration of KCF18 increased, the measured response for the binding of KCF18 to TNF-α also increased, indicating that binding was concentration dependent. (**B**) For the steady–state interaction, a binding isotherm was generated to determine the equilibrium K_D_ and R_max_ for the binding of KCF18 to cytokine TNF-α, which were found to be 60.9 μM and 197.1 RU, respectively. (**C**) KCF18 was injected over IL-6 immobilized on the CM5 sensor chip. The measured response for the binding of KCF18 to IL-6 also increased with the concentration of KCF18, similar to the profile of the binding of KCF18 to TNF-α. (**D**) The kinetic analysis of binding isotherm was also performed to determine the equilibrium dissociation K_D_ and R_max_ for KCF18 binding to cytokine IL-6, which were found to be 111.5 μM and 210.3 RU, respectively.

To confirm whether KCF18 binds to the three cytokines, the composite peptide KCF18 was synthesized for SPR measurement by using a Biacore T200 instrument. SPR sensorgrams revealed a positive change in RUs. This finding revealed that KCF18 could bind to the cytokines (TNF-α and IL-6) immobilized on the CM5 sensor chip (Fig. 3). The measured response for the binding of KCF18 to TNF-α also increased when the concentration of KCF18 increased, indicating that binding occurred in a concentration-dependent manner (Fig. 3A). When KCF18 was injected, KCF18 rapidly bound to TNF-α, and the curve plateaued after a few seconds. Thereafter, KCF18 dissociated rapidly during the rinsing of the chip with buffer. For a steady–state interaction, the binding isotherm was generated to determine the equilibrium dissociation constant K_D_ (60.9 μM) and R_max_ (197.1 RU) for the binding of KCF18 to TNF-α (Fig. 3B). The measured response for the binding of KCF18 to IL-6 also increased with the KCF18 concentration, similar to the profile for the binding of KCF18 to TNF-α, as shown in Fig. 3C. The kinetic analysis of the binding isotherm was also performed to determine the equilibrium dissociation K_D_ (111.5 μM) and R_max_ (210.3 RU) for the binding of KCF18 to the cytokine IL-6 (Fig. 3D). In addition, the measured response for the truncated peptide SEM18 to TNF-α increased with the SEM18 concentration, similar to the profile for the binding of KCF18 to TNF-α, as shown in Supporting Figure S3. The kinetic analysis of the binding isotherm was also performed to determine the equilibrium dissociation K_D_ (68.3 μM) and R_max_ (8.2 RU) for the binding of SEM18 to the cytokine TNF-α. Because it was extremely difficult to immobilize IL-1β on the sensor chip, the interaction between KCF18 and IL-1β was not measured through SPR detection but was confirmed through cellular assays and the animal model.

### Composite peptide binding to the cytokines for inhibition of cytokines induced monocyte binding and transmigration to endothelial cells

**Figure 4:**
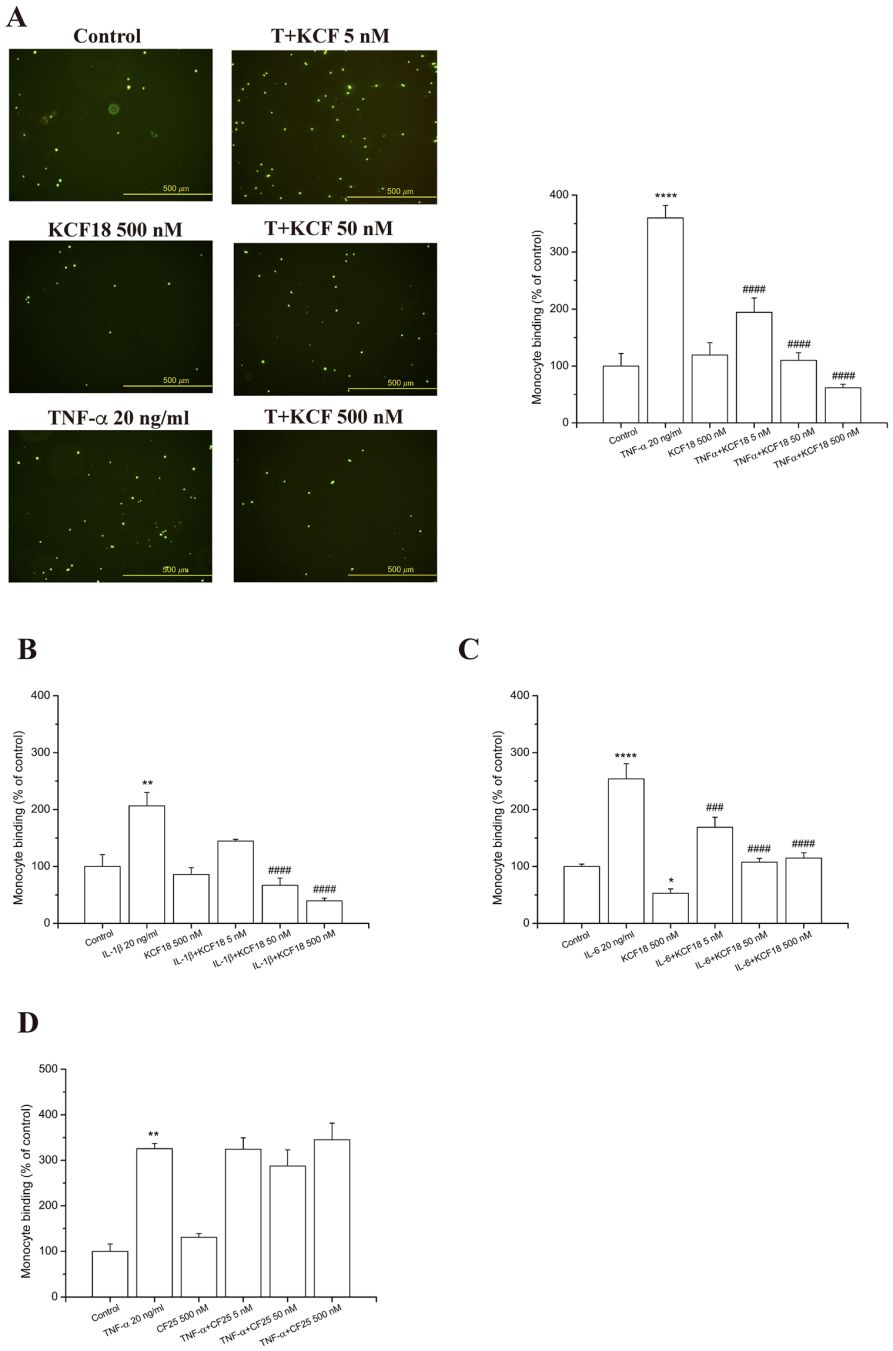
KCF18 inhibits various cytokine-induced monocyte adhesion to HMEC-1. HMEC-1 cells were pretreated with various concentrations of KCF18 or (**D**) peptide CF25 for 1 hour and were then stimulated with 20 ng/mL (**A**) TNF-α, (B) IL-1β, or (**C**) IL-6 for 18 hours. Adhesion of fluorescent THP-1 cells was photographed by fluorescent microscopy, and fluorescence intensity was calculated. “Control” cells were only incubated with the culture medium (without peptides). Values are mean ± SD from three independent experiments. *P < 0.05, **P < 0.01, ***P < 0.001 and ****P < 0.0001 as compared with control; ^#^P < 0.05, ^##^P < 0.01, ^###^P < 0.001 and ^####^P < 0.0001 as compared with cells stimulated with cytokines in the absence of peptides.

**Figure 5:**
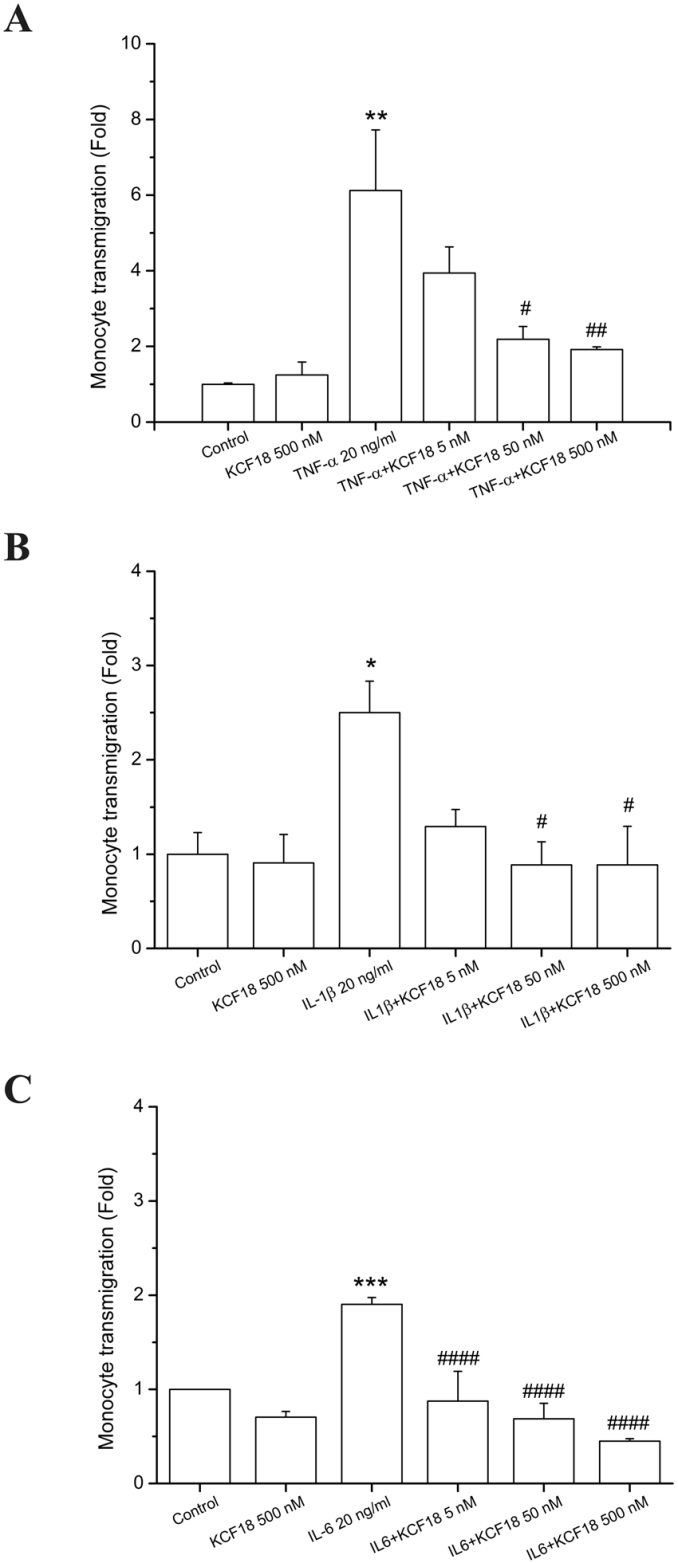
KCF18 inhibits cytokine-induced transmigration of monocytes. HMEC-1 cells were pretreated with various concentrations of KCF18 for 1 hour and then stimulated with 20 ng/mL (**A**) TNF-α, (**B**) IL-1β, or (**C**) IL-6 for 18 hours; thereafter, THP-1 cells were allowed to transmigrate through the HMEC-1 monolayer. Values are mean ± SD from three independent experiments. *P < 0.05, **P < 0.01, ***P < 0.001 and ****P < 0.0001 as compared with control; ^#^P < 0.05, ^##^P < 0.01, ^###^P < 0.001 and ^####^P < 0.0001 as compared with cells stimulated with cytokines in the absence of KCF18.

To confirm that the *in silico* designed composite peptide binds to the proinflammatory cytokines, peptides at different concentrations (500 nM, 50 nM, and 5 nM) were incubated with the three cytokines to examine whether the peptide inhibits the adherence of THP-1 cells (human acute monocytic leukemia cell line) to cytokine-activated human microvascular endothelial cells (HMEC-1). The activation of HMEC-1 by TNF-α, IL-1β and IL-6 leaded to important enhancement in the count of stuck THP-1 on the HMEC-1 monolayer. Meanwhile, the pretreatment of HMEC-1 with KCF18 repressed the count of THP-1 cells sticking to various cytokine-treated HMEC-1 cells, with an almost 100% (*P* < 0.001) decrease (Fig. 4). Thus, KCF18 could inhibit cytokine-induced monocyte binding. This finding indicated that the peptide suppressed the association between the cytokines with the cell surface receptors. To verify whether the suppression is caused by the peptide KCF18, a random peptide with 25 amino acids (CF25; ^1^CPLNGSTVYGHLRHCLSCSGTMVKF^25^) and a truncated peptide SEM18 derived from TNFR1 were synthesized for monocyte binding assays. The results showed that the nonrelated peptide CF25 could not repress cytokine-increased monocyte binding to HMEC-1 cells and peptide SEM18 could only reduce TNF-α-induced monocyte binding, but not cytokines IL-1β, and IL-6 (Fig. 4D and Supporting Figure S4). Furthermore, transmigration assays were performed to examine whether KCF18 inhibits cytokine-induced monocyte transmigration. The numbers of transmigrating THP-1 cells were significantly increased when endothelial cells were cultured with the cytokines (TNF-α, IL-1β, and IL-6) compared with the migration in the absence of cytokines. Treatment with the designed peptide KCF18 could reduce the number of transmigrating THP-1 cells induced by the cytokines (Fig. 5). The result of the transwell assay was similar to that of the monocyte binding assay. Thus, KCF18 could inhibit the binding of the cytokines to their receptors on the cell surface. Therefore, the three cytokines could not transmit the inflammatory signal to cells for regulating immune responses.

To evaluate whether the inhibitory effects of the composite peptide KCF18 on THP-1 binding and endothelial transmigration were as a result of the cytotoxic effects on endothelial cells, HMEC-1 cells were treated with various concentrations of KCF18. The viability of HMEC-1 cells was assessed by the WST-1 assay after 24 and 48 hours treatment. As described in Supporting Figure S5, no important discrepancy was noticed in cell viability among control cells and peptide-treated cells. This result suggested that endothelial cytotoxicity is not the reason for the repression of THP-1 adherence and endothelial transmigration by KCF18.

### Inhibitory effects of composite peptide KCF18 on inflammatory cytokine-induced TNF-α transcription

**Figure 6:**
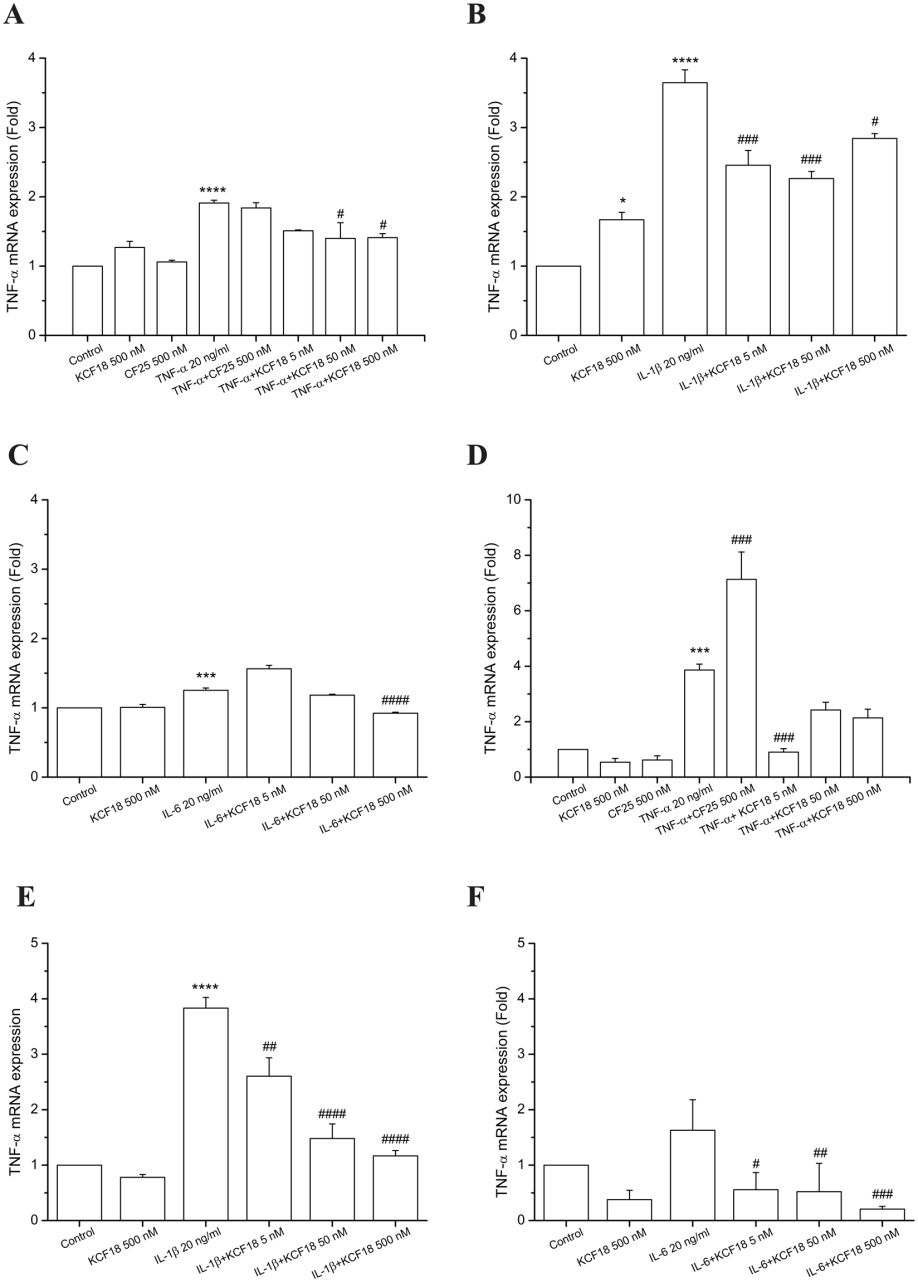
Peptides KCF18 and CF25 affect the expression of cytokine-induced TNF-α in HMEC-1 and THP-1 cells. TNF-α mRNA levels induced by TNF-α (**A** and **D**), IL-1β (**B** and **E**), or IL-6 (**C** and **F**) in HMEC-1 (**A–C**) and THP-1 (**D–F**) were determined using qPCR assays, as described in Materials and Methods. GAPDH cDNA was used as an internal control. Values are mean ± SD of mRNA levels relative to those for GAPDH from three independent experiments. *P < 0.05, **P < 0.01, ***P < 0.001 and ****P < 0.0001 as compared with control; ^#^P < 0.05, ^##^P < 0.01, ^###^P < 0.001 and ^####^P < 0.0001 as compared with cells stimulated with cytokines in the absence of the peptides.

We used qPCR assays to quantify the effects of KCF18 on TNF-α-, IL-6-, and IL-1β-induced TNF-α mRNA expression. Compared with control cells, TNF-α mRNA expression levels were increased in HMEC-1 (Fig. 6A–C) and THP-1 cells (Fig. 6D–F) cultured with TNF-α. The negative control peptide CF25 had no inhibitory effect on TNF-α-induced TNF-α mRNA expression (Fig. 6A). However, peptide KCF18 significantly reduced TNF-α-mediated TNF-α mRNA expression. In addition, no significant difference was observed in mRNA expression levels between peptide-treated cells and control cells (Fig. 6A). Similarly, KCF18 suppressed IL-6- and IL-1β-induced TNF-α mRNA expression. This finding indicated that KCF18 interfered with the binding of these cytokines to their receptors and further reduced downstream TNF-α mRNA transcription (Fig. 6B–F).

### Anti-inflammatory effects of KCF18 in a peritonitis model *in vivo*

**Figure 7:**
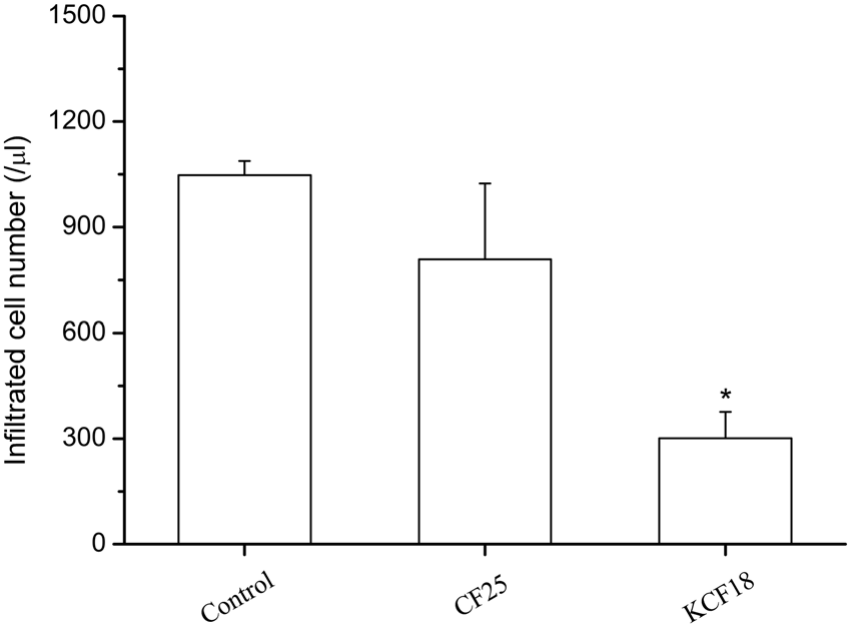
KCF18 alleviates peritonitis. Cell counts from peritoneal fluid 24 hours after intraperitoneal injection of thioglycollate in mice are shown. Comparisons of KCF18 with CF25. Values are mean ± SEM (n = 6 for control vs. n = 7 for the peptide CF25 and n = 5 for KCF18 treatment). *P < 0.05 as compared with control.

Neutrophils are rapidly mobilized from the bone marrow into the blood during acute inflammatory reactions. Intraperitoneal injection of thioglycollate elicits a robust influx of neutrophils into peritoneal cavity^50,51^. We assessed the effects of KCF18 on inflammatory cell recruitment by using a thioglycollate-induced acute peritonitis model. At 24 hours after thioglycollate stimulation, elicited inflammatory cells were detected in the peritoneal cavity. Administration of KCF18 at 4 hours after thioglycollate stimulation significantly reduced total white blood cell infiltration in the peritoneal cavity. However, the negative control peptide CF25 had no potential anti-inflammatory effect (Fig. 7). As estimated, the influence of mKCF18 on reducing of WBC infiltration is worse than KCF18 peptide (Supporting Figure S9).

## Discussion

The first step in the process of vascular inflammation was the adhesion of monocytes to the endothelium. Subsequently, the monocytes infiltrated the endothelial wall and differentiated into macrophages. This critical step was modulated by the interaction between monocytes and the surface molecules of endothelial cells^52,53^. During the inflammatory response, TNF-α was initially released, regulating IL-6 and IL-8 levels. Similar to TNF-α, IL-1β, one of the most important proinflammatory mediators of local acute inflammation, is an alarm-phase cytokine that can elicit numerous clinical features of inflammation. Studies have suggested that the inhibition of TNF-α, IL-6, and IL-1β tends to alleviate inflammation exacerbation.

**Figure 8:**
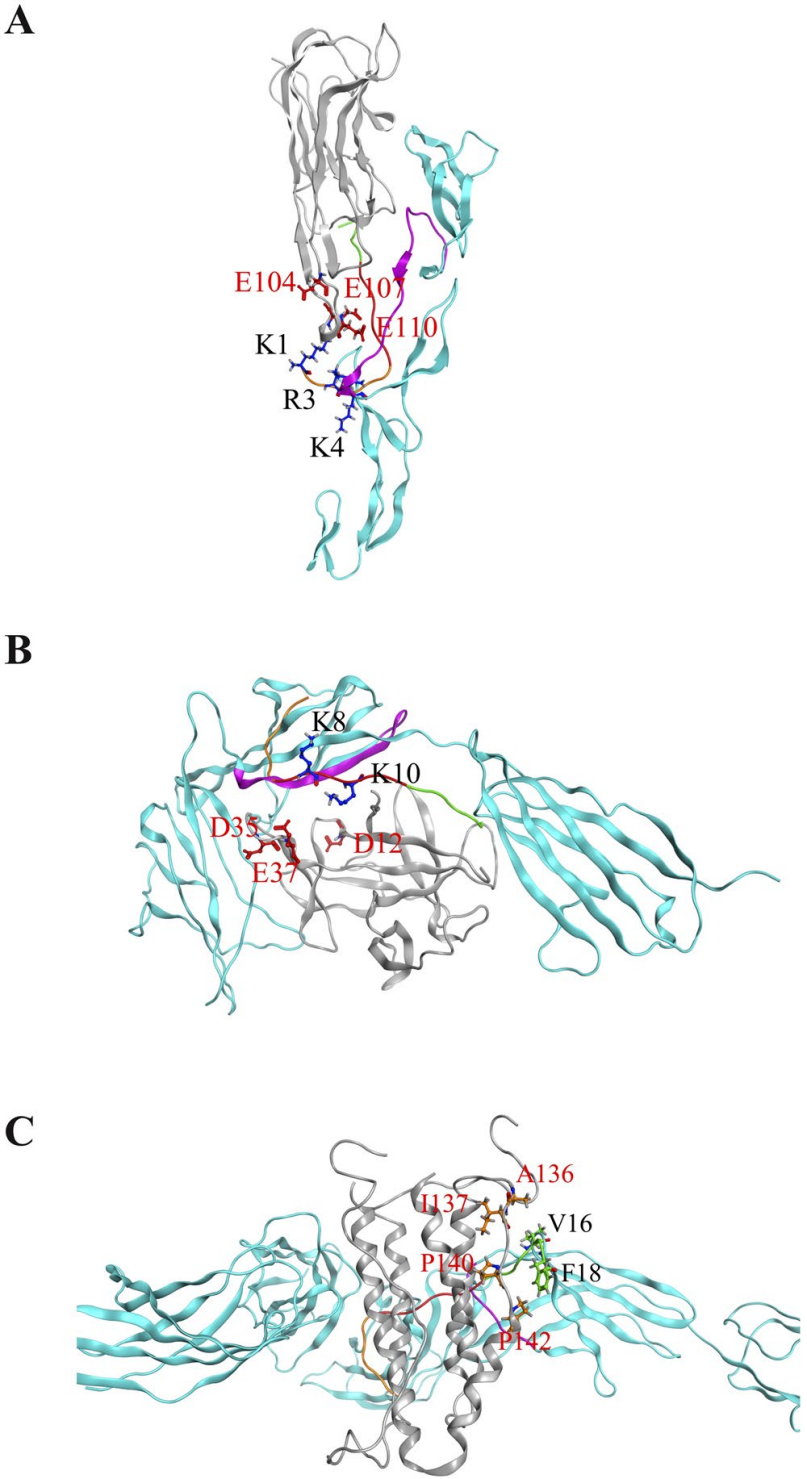
Superposition of peptide KCF18-cytokine system and receptor-cytokine system. (**A**) KCF18-TNF-α and TNF-α-TNFR1 (**B**) KCF18-IL-1β and IL-1β-IL1R (**C**) KCF18-IL-6 and IL-6-IL6R. Gray color is cytokine, cyan color is receptor, purple color is the region selected from receptor to compose the peptide KCF18, and peptide KCF18 is composed of three color short peptide (orange: TNFR1; red: IL1R; green: IL6R).

Therefore, in this study, according to *in silico* analysis of molecular docking and MM/PBSA binding free energy calculations, a potential composite peptide was designed to bind these proinflammatory cytokines simultaneously. The designed peptide KCF18 was composed of 18 amino acids derived from the receptors of the three proinflammatory cytokines. Regarding the composition of the peptide, the six N-terminal amino acids (KCRKEM) from TNFR1 were more positively charged, the six middle amino acids (FKQKLP) from IL-1R were more hydrophobic and positively charged, and the six C-terminal amino acids (YSTVYF) from the IL-6 receptor were neutral (Fig. 1). Comparison of the KCF18–cytokine theoretical structures with the cytokine–receptor structures suggests that the designed peptide KCF18 binds to the proinflammatory cytokines similar to the original binding sites (Fig. 2 and Supporting Figure S1) by superposing the KCF18–cytokine and receptor–cytokine complexes (Fig. 8). For example, the theoretical calculations suggest that the N-terminal region of KCF18 dominantly bound to the negatively charged region of TNF-α (E104, E107, and E110) through electrostatic interactions. The positively charged residues of KCF18 were bound to the negatively charged residues of IL-1β (D12, D35 and E37); thus, the predicted binding site was near the original binding site. This binding pattern may result from the influence of other residues of KCF18. In the KCF18–IL-6 complex, both electrostatic and hydrophobic interactions were dominant. KCF18 bound to each proinflammatory cytokine through specific residues derived from the cognate receptor, and the other residues of KCF18 enhanced the binding. The MM/PBSA binding free energy calculations showed that the electrostatic interactions were dominant in the binding of the peptide to these proinflammatory cytokines, whereas VDW interactions were considerably minor, which is consistent with our docking results (Supporting Figure S2) and a previously proposed peptide for inhibiting LPS binding^54–56^. Moreover, *in vitro* SPR measurements also confirmed that the designed peptide KCF18 bound to TNF-α and IL-6. Similar to the SPR measurements, the binding free energy for the binding of KCF18 to TNF-α was lower than that for the binding of KCF18 to IL-6. This result showed that KCF18 had a higher binding affinity with TNF-α than with IL-6 (Fig. 3 and Supporting Figure S2). In addition, alanine scanning analysis was also performed for some positively charged resides (K1, R3, K4, K8, and K10) to show that mutant residues R3A, K8A and K10A of peptide KCF18 will increase the binding free energy (ΔΔG = 123.1, 35.3, and 82.0 kJ/mol) and decline the binding affinity (Supporting Table S1). This was also confirmed in our cellular experiments treated with the mutant peptide mKCF18 (KCAKEMFAQKLPYSAVYF) (Supporting Figures S6 and S7).

In the current study, we demonstrated that the anti-inflammatory effect of KCF18 is attributed to the reduction of cytokine–receptor interactions on endothelial cells and monocytes. In addition, the decline in cytokine–receptor interactions might suppress cytokine mRNA expression and cytokine production in endothelial cells and monocytes. Treatment with KCF18 suppressed TNF-α mRNA expression following cytokines TNF-α, IL-1β and IL-6 stimulation (Fig. 6). To assess the efficacy of KCF18, we performed the RT-qPCR in THP-1 and HMEC-1 cells and ELISA assay for detecting TNF-α secretion including a TNF-α blocking antibody (R&D systems, USA)^57–59^ as an additional condition in their experiments. KCF18 peptide decreased TNF-α induced TNF-α mRNA expression in a dose-dependent manner in both cells (Supporting Figure S6). The scramble peptide, mKCF18, had no inhibitory effect on TNF-α induced TNF-α mRNA expression in both cells. As prediction, the TNF-α blocking antibody inhibited TNF-α induced TNF-α mRNA expression in a concentration (1 μg/ml) similar to the peptide concentration of 500 nM. Anti-IgG, negative control of TNF-α blocking antibody, did not decline TNF-α induced TNF-α mRNA expression (Supporting Figure S6). We further analyze whether the KCF18 could suppress IL-1β induced TNF-α or TNF-α induced IL-1β expressions by ELISA assay (Supporting Figure S7). The results showed that 20 ng/ml IL-1β induces 2 fold secretion of TNF-α in the THP-1 cells. Consistent with the RT-qPCR result, pretreatment of KCF18 decreased the TNF-α expressions. However, mKCF18 could not inhibit IL-1β induced TNF-α expression. TNF-α blocking antibody dramatically suppresses IL-1β induced TNF-α expression. On the other hand, KCF18 also decreased TNF-α induced IL-1β expressions in a dose-dependent manner even though the expression levels of IL-1β were low. TNF-α blocking antibody suppressed TNF-α induced IL-1β expression. Since TNF-α blocking antibody captures the TNF-α in the conditioned medium, it may interfere the detection of TNF-α by ELISA (Supporting Figure S7). In summary, indeed, KCF18 expresses the ability to reduce cytokines induced TNF-α mRNA and protein expression. Our results also revealed that treatment with the peptide reduced the adherence of THP-1 to cytokine-activated endothelial cells and suppressed endothelial transmigration of THP-1 (Figs 4 and 5). We also assessed the ability of KCF18 to inhibit TNF-α-mediated p65 nuclear translocation and endothelial ICAM-1 expression (Supporting Figure S8). KCF18 reduced TNF-α-induced p65 nuclear translocation by Western blot. KCF18 also decreased TNF-α-mediated endothelial ICAM-1 expression (Supporting Figure S8). Intraperitoneal injection of thioglycollate elicits a robust influx of neutrophils into the peritoneal cavity. The trafficking of the cells is believed to be mediated by chemokines CXCL1, CXCL2, and CXCL8^60,61^. Thus, this model can be used to test the inhibitory effect of KCF18 on the chemoattractive activities of these chemokines, which induce neutrophil migration. In our animal model of peritonitis, we observed the inhibition of white blood cell infiltration, which confirmed the anti-inflammatory properties of KCF18 (Fig. 7 and Supporting Figure S9).

Multiple anti-inflammatory immunomodulatory therapies have been developed to ameliorate proinflammation following bacterial infection. However, these immunomodulatory agents, such as inhibitors of endotoxin, TNF-α, IL-1β, and Toll receptor 4 (TLR4) (e.g., TAK-242, a small-molecule inhibitor of TLR4, and eritoran, a specific antagonist of MD2-TLR4)^62–65^, have been proven to be unsuccessful in clinical trials. Therefore, novel therapeutic approaches that decrease inflammatory responses should be developed, and researchers should expand the focus beyond agents that only treat the initial infectious agent to those that augment the host’s immune response to cytokines. Peptide inhibitors might be more specific than small-molecule inhibitors. However, the main problem with inhibitory peptides is their delivery to cells. No such problem was observed for our system, because this peptide was designed to interfere with the cytokine–receptor interaction on the cell surface. The other problem with peptides is their stability, namely low bioavailability and metabolic liability, in live organisms. In this regard, our results showed that KCF18, which bound to multiple cytokines, prevented leukocyte infiltration in the peritonitis model (Fig. 7 and Supporting Figure S9), which might be valuable. It has been observed that although the presence of the receptor may be a prerequisite for a biological effect, the number of receptors may not be correlated with the magnitude of the response^66,67^. This may be the reason why physiological cell responses (monocyte adhesions and transmigration) do not coincide with the results of molecular biological assays (qPCR).

Taken together, our data revealed a new role of this peptide in targeting the receptors of three cytokines, affecting the regulation of inflammatory responses *in vitro* and *in vivo*. The computational simulations agreed with our experimental results, showing that it is a useful tool for the development of anti-inflammatory peptides. Our results suggested that peptide-mediated inhibition of multiple cytokine-induced immune responses may provide a unique opportunity to develop an urgently needed therapeutic intervention strategy for inflammation-related diseases, and the peptide may serve as a novel immunomodulatory therapeutic agent that can be utilized for the design and discovery of new therapeutic drugs to treat inflammation.

## Materials and Methods

### The protein structures of cytokines

The cytokines used in this study for molecular docking and MD simulations were selected from protein data bank (http://www.rcsb.org/pdb/home/home.do) with PDB codes 1TNF for TNF-α, 1ITB for IL-1β, and 2IL6 for IL-6.

### Construction of full-length IL-6

Because the resolved NMR structure of human IL-6 lacked 20 amino acids at its N-terminus (PDB code: 2IL6) which may affect the binding to its cognate receptor, the N-terminus of human IL-6 was created by homology modeling from Phyre2 web server (http://www.sbg.bio.ic.ac.uk/phyre2/)^68^. The modeled N-terminus of human IL-6 (residues 6–20) using the NMR structure of mouse IL-6 (PDB code: 2L3Y)^69^ as a template was then connected with the original NMR structure of human IL-6 (PDB code: 2IL6)^36^ to form a full-length human IL-6 by using the MOE2015.10 software (Molecular Operating Environment, http://www.chemcomp.com). The structure was then performed 50 ns molecular dynamics (MD) simulations for equilibration.

### Molecular docking

All the molecular dockings, such as the designed peptide KCF18 docked with the cytokines TNF-α, IL-1β, and IL-6, and the TNF-α redocked with receptor TNFR1, were performed by using “dock proteins protocol” (ZDOCK) from BIOVIA Discovery Studio 3.5. The ZDOCK protocol was used to conduct the rigid-body docking of two protein structures as well as clustering the poses according to the ligand position using a Fast Fourier Transformation (FFT) to perform an exhaustive six-dimensional search in the translational and rotational space between the two molecules. The rotational search sampling grid is used as a 6° grid which samples a total of 54000 docked poses per system. In the following, 200 poses were selected to run RDOCK to refine the protein structures. RDOCK algorithm uses a CHARMm-based minimization procedure for scoring and refinement the complexes from ZDOCK by removing clashes, optimizing polar interactions and charge interactions. The RDOCK scores are defined as the summation of the electrostatic energy and the desolvation energy. Finally, the preferable poses will be selected from the lowest RDOCK scores for further MD simulations.

### Molecular dynamics (MD) simulations

The GROMACS-4.5.5 software was used to perform all MD simulations with Gromos96 (ffG45a3) force field and an integration step size of 2 fs. The MD simulation protocol was as followed, after energy minimization and equilibration, 200 ns production runs were carried out without any constraint on the complex structure. The simulations were conducted in the *NPT* ensemble employing the velocity-rescaling thermostat at constant temperature 310 K, and 1 bar. The temperature of the complex protein, and the solvent were separately coupled with a coupling time of 0.1 ps. The systems were neutralized with sodium and chloride ions generating 0.15 M NaCl solution. Isotropic pressure coupling was applied with a coupling time of 0.1 ps and a compressibility of 4.5 × 10^−5^ bar^−1^ for the x-, y-, and z-direction. Long-range electrostatics is calculated using the particle-mesh Ewald (PME) summation algorithm with grid dimensions of 0.12 nm and interpolation order 4. Lennard-Jones and short-range Coulomb interactions were cut off at 1.4 and 1.0 nm, respectively. The following equilibration protocol was used: (i) the temperature was gradually increased from 100 K to 200 K and 310 K. The system was run for 500 ps for each temperature. During these simulations, the complex structure remained fully restraint (k = 1000 kJ mol^−1^ nm^−2^). (ii) At 310 K, the restraints kept on the complex structure via the force constant k, were released in 3 steps from k = 500 kJ mol^−1^nm^−2^ to k = 250 kJ mol^−1^nm^−2^, and finally k = 100 kJ mol^−1^nm^−2^. Each step was run for 2.0 ns. All complexes were run for 200 ns without any constraint on the complex structures.

### MM/PBSA binding free energy calculations

To determine the most stable peptide-cytokine complexes, MM/PBSA binding free energy calculations (Δ*G*_*bind*_) were used, which was according to the snapshots extracted from the single trajectory of the complex (single trajectory method)^70,71^. The cytokine and the peptide are assumed to behave similarly in the binding, which is reasonable and adopted for our simulations. For more detailed settings of the free energy calculations, please refer to the previous studies^48,72,73^. Finally, to calculate all the energy terms, a total of 300 snapshots extracted from the last 30 ns stable MD trajectory (170−200 ns) per system were performed. The binding free energy calculating tool in Gromacs-4.5.5 (GMXAPBS tools) is supported from Musco group^73^.

### Recombinant proteins and peptide synthesis

The recombinant proteins of TNF-α (catalog^#^1130-01), IL-1β (catalog^#^1110-01B), and IL-6 (catalog^#^1110-06) were purchased from the GOLDBIO.COM, USA. Based on the *in silico* analysis of this study, the designed peptide (KCF18), a truncated peptide (SEM18) derived from TNFR1, a mutant peptide (mKCF18), and a random peptide for negative control (CF25) were chemically synthesized by GeneMark (GMbiolab Co., Ltd., Taiwan) with a solid phase methodology for *in vitro* and *in vivo* experimental confirmation.

### Surface Plasmon Resonance (SPR) measurements

The peptide KCF18 predicted from molecular docking and MM/PBSA binding free energy calculations were synthesized to confirm whether the peptide can bind to the pro-inflammatory cytokines by using SPR measurements. A Biacore T200 workstation (GE Healthcare, USA) was used for SPR detection. These cytokines (250 μg/ml TNF-α and 400 μg/ml IL-6) were diluted into 10 mM sodium acetate buffer at pH 4.0 and immobilized onto a CM5 sensor chip using amine coupling (EDC-NHS) for 210 secs at a flow rate of 10 μl/min. Approximately 5000 and 2000 response units (RU) of cytokines TNF-α and IL-6 were immobilized on the sensor chip, respectively. The flow channel-1 also treated the EDC-NHS as a reference to reduce the non-specific binding between dextran and peptides. The single cycle kinetics method was utilized to calculate the binding affinity of cytokines to the designed peptide KCF18 by flowing five concentrations (2 μM, 10 μM, 25 μM, 50 μM, and 100 μM) over the chip sequentially at a flow rate of 10 μl/min at 25 °C. The complexes were allowed to associate for 180 secs and then to dissociate for 90 secs. Surfaces were regenerated with an injection of 2 mM MgCl_2_ at a flow rate of 30 µl/min for 30 secs. A monovalent binding model was used to generate and fit the equilibrium binding curves for each peptide to determine the K_D_ of the binding of peptide KCF18 with the pro-inflammatory cytokines.

### Cell Culture

Human micro-vascular endothelial cells-1 (HMEC-1, from American Type Culture Collection Manassas, USA) was cultured in MCDB 131 medium (Sigma, USA) with 15% fetal bovine serum (FBS; Sigma, USA). The medium was supplemented with 1% L-glutamine, 500 µg/ml streptomycin (Biowest, USA), 500 units/ml penicillin (Biowest, USA), 1 μg/ml hydrocortisone (Sigma, USA) and 10 ng/ml Epidermal Growth Factor (ProSpec, USA). The human acute monocytic leukemia cells (THP-1 cells) obtained from the Bioresource Collection and Research Center (Hsinchu, Taiwan) were cultivated in the RPMI-1640 medium (Invitrogen, USA) with 10% FBS. THP-1 were differentiated to macrophage by 40 μM Phorbol 12-myristate 13-acetate (PMA; Sigma, USA) for 24 hours before peptides treatment.

### Cell cytotoxic assay

To assess whether the peptide KCF18 is toxic to the endothelial cells, 1 × 10^4^ cells were placed into 96-well micro-plates and incubated for 2 hours for adherence. After adhesion, the medium with various concentrations of peptides (0.5 μM to 5 μM) were added to the wells. After 24 and 48 hours, the HMEC-1 cells were washed with Hank**’**s balanced salt solution and the survivals of HMEC-1 were detected by WST-1 assay kit (Roche, USA). The experiment was assessed in quadruplicate and repeated three times.

### Monocyte binding assay

To perform the test of monocyte adherence to endothelial cells monolayers, the HMEC-1 cell monolayers were cultivated on 24-well plates under serum-starvation condition prior to the monocyte binding assay. The peptides were added to the HMEC-1 cells for one hour prior to the activation by 20 ng/ml TNF-α (Sigma, USA), IL-1β (GOLDBIO, USA) and IL-6 (GOLDBIO, USA) for 18 hours. The THP-1 monocytes pre-labeled with 5 mM Calcein-AM (Invitrogen, USA) for 30 minutes in RPMI 1640. After the monolayers were washed twice to eliminate TNF-α, IL-1β and IL-6, the pre-labeled THP-1 cells (5 × 10^5^ cells/well) were incubated with HMEC-1 and cultivated for 30 minutes at 37 °C. The non-stuck THP-1 cells were removed from monolayers by Hank**’**s balanced salt solution five times. The adhered monocytes were detected and photographed (five images per well) by fluorescent microscopy and the amount of stuck THP-1 cells were counted by the software AlphaImager 2200 (Alpha Innotech, USA).

### Transmigration assay

To perform the transmigration of THP-1 cells through HMEC-1, the experiment was designed in a Trans-well system (BD Biascences, USA). The HMEC-1 cells were cultivated on trans-well (8 µm pore sizes of polycarbonate membranes) membranes. Then the peptides were added to the cells for one hour prior to stimulation of 20 ng/ml TNF-α for 18 hours. THP-1 cells (5 × 10^5^ cells/50 µl/well) in RPMI1640 were put into the upper chamber of trans-well inserts. 20 ng/ml TNF-α, new design peptide (KCF18) (0.5 µM) were put into the lower chamber for the initiation of transmigration. After six hours of culture, the THP-1 cells across to the lower chamber were collected. Then the numbers of cell were counted by using microscopy. All experiments were performed in at least two times independently.

### RNA isolation and quantitative polymerase chain reaction (qPCR) assays

Cells were grown to confluence in 6-cm^2^ culture plates. Peptides were incubated with TNF-α (20 ng/ml) for 1 h and then treated the cells for 4 h. Total RNA was isolated using Trizol Reagent (Invitrogen), according to the manufacturer’s suggested protocol. An aliquot (5 µg) of purified RNA was reverse transcribed into first-strand complementary DNA (cDNA) with a 2720 Thermal Cycler (Applied Biosystems, Grand Island, NY, USA), 200 U/μl M-MLV reverse-transcriptase (Invitrogen) and 0.5 mg/μl oligo(dT)-adapter primers (Invitrogen) in a 20 μl reaction mixture. The qPCR assays for TNF-α, IL-6, and glyceraldehyde 3-phosphate dehydrogenase (GAPDH) were performed with a Roche LightCycler 480 System (Roche, Indianapolis, IN, USA) and iQ SYBR Green Supermix (Bio-Rad, Hercules, CA, USA). The oligonucleotide primers used were specific for TNF-α (5′-AGG GAC CTC TCT CTA ATC AG-3′ and 5′-TGG GAG TAG ATGAGG TAC AG-3′), IL-6 (5′-GCC GCC CCA CAC AGA CA-3′ and 5′-CCGTCG AGG ATG TAC CGA AT-3′), and GAPDH (5′-ACG GAT TTG GTCGTA TTG GG-3′ and 5′-TGA TTT TGG AGG GAT CTC GC-3′). Thermal cycling conditions involved an initial denaturation step at 95 °C followed by 35 amplification cycles (15 s at 95 °C and 20 s at 60 °C) and subsequent melt curve analysis (72−98 °C). Quantitation of gene expression was conducted relative to GAPDH expression levels.

### Mouse model of peritonitis

The animal experiment was used 8- to 10-week-old male BABL/c mice to be the animal model. These mice were purchased from the National Laboratory Animal Breeding and Research Center, Taipei. The mice were housed in a temperature-controlled, light-cycled facility. Peritonitis was induced by an intraperitoneal injection of 4% (w/v) thioglycollate in 1 ml of sterile saline (Sigma–Aldrich, USA). Treatment with saline or peptides (5 mg/kg) was performed 4 h after the administration of thioglycollate by intravenous injection. At 24 h after thioglycollate injection, mice were killed by exposure to CO_2_, and 5 ml of HBSS was injected into the peritoneal cavity. Cells were obtained by aspirating peritoneal lavage. Differential cell counts were determined using a Hematology Analyzer (KX-21N; Sysmex, USA).

### Ethics Statement

This study was carried out in strict accordance with the recommendations in the Guide for the Care and Use of Laboratory Animals of the National Institutes of Health. This animal experiment performed by Dr. Shih-Yi Peng was allowed and approved by Tzu Chi University Institutional Animal Care and Use Committee (Permit Number: 104077).

### Statistical analysis

Results are expressed as the means ± s.e.m from at least three independent experiments. Differences between groups were assessed by one-way analysis of variance (ANOVA) and Tukey’s test of post-hoc analysis. A P-value less than 0.05 was considered statistically significant.

## Electronic supplementary material

Supplementary information
