# Supplementary material for: A potential peptide derived from cytokine receptors can bind proinflammatory cytokines as a therapeutic strategy for anti-inflammation
Source: Sci Rep. 2019 Feb 19;9:2317. doi: 10.1038/s41598-018-36492-z (PMC6381106; doi:10.1038/s41598-018-36492-z)
Supplement: Supplementary file 1 — Supplementary information [file 41598_2018_36492_MOESM1_ESM.pdf]

Supplementary information

A potential peptide derived from cytokine receptors can bind proinflammatory cytokines as a therapeutic strategy for anti-inflammation

Shinn-Jong Jiang[1,$], Pei-I Tsai[2,$], Shih-Yi Peng[1], Chun-Chun Chang[3,4], Yi Chung[1], Hao-Hsiang Tsao[1], Hsin-Ting Huang[1], San-Yuan Chen[2] and Hao-Jen Hsu[1,5*]

[1]*Department of Biochemistry, School of Medicine, Tzu Chi University, Hualien 97004, Taiwan*

[2]*Department of Materials Science and Engineering, National Chiao-Tung University, Hsinchu 30010, Taiwan*

[3]*Institute of Medical Sciences, Tzu Chi University, Hualien 97004, Taiwan*

[4]*Department of Laboratory Medicine, Tzu Chi Medical Center, Hualien 97004, Taiwan*

[5]*Department of Life Sciences, Tzu Chi University, Hualien 97004, Taiwan*

Corresponding Author

*Hao-Jen Hsu: E-mail address: hjhsu32@mail.tcu.edu.tw

¶These authors contributed equally to this work.

S1A

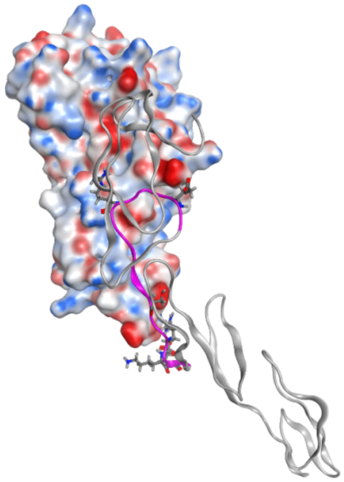

S1B

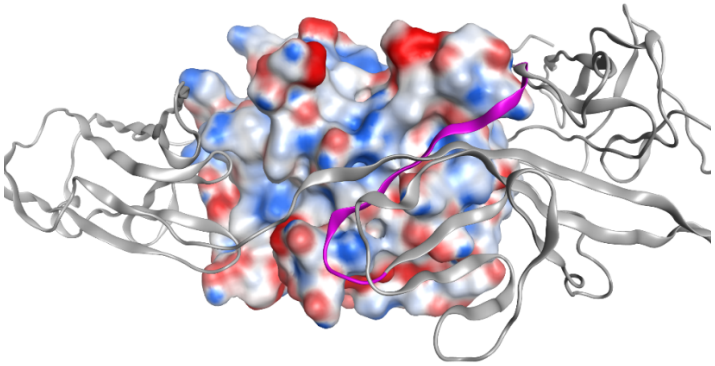

S1C

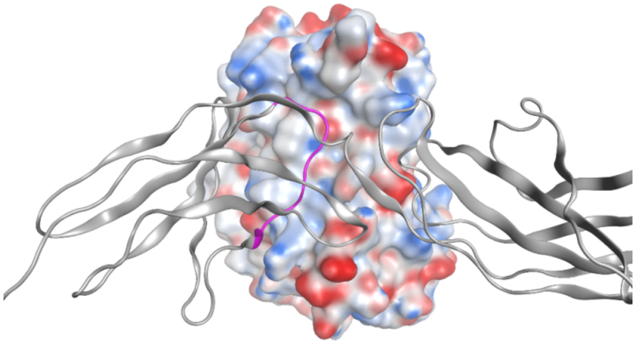

Figure S1: Surface charge distributions of pro-inflammatory cytokine/receptor complex structures based on Poisson-Boltzmann equation. Blue color corresponds to positive and red color to negative electrostatic potential. Cytokine receptors are represented as ribbon structure colored gray. The pink color loop is the determined binding region from the receptor. (A) The preferable pose of cytokine TNF-α binding to receptor TNFR1. (B) The ectodomain of interleukin-1 receptor (IL-1R) complexed with cytokine IL-1β (PDB code: 1ITB) (C) The preferable pose of cytokine full-length IL-6 binding to ectodomain of receptor.

S2A

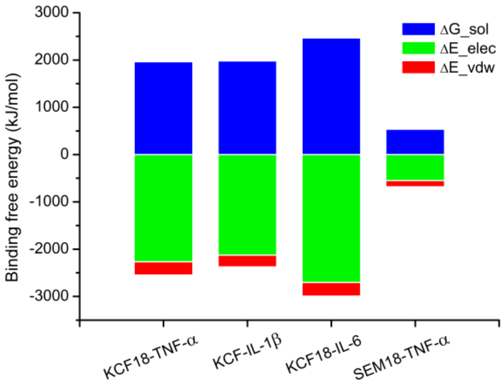

S2B

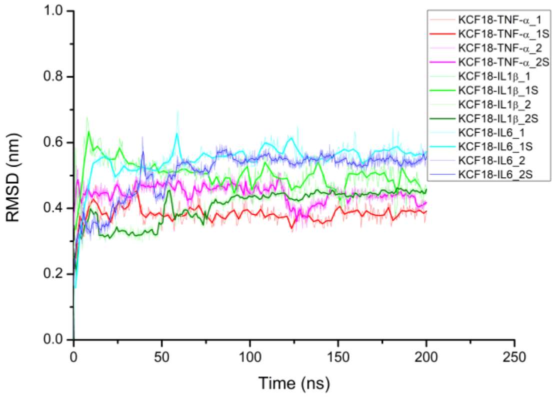

S2C

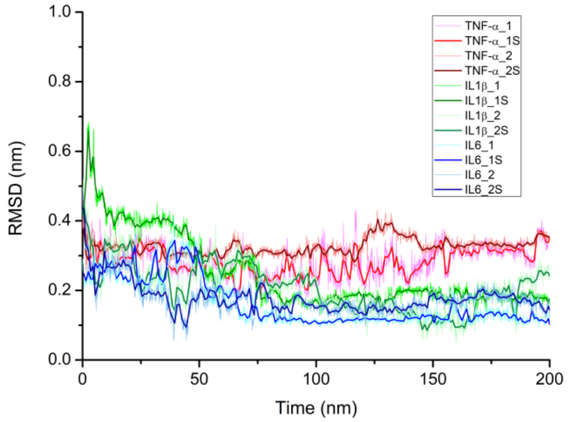

Figure S2: The MM/PBSA binding free energy calculations for designed peptide KCF18 and truncated peptide SEM18 binding to pro-inflammatory cytokines and RMSD profiles of the complex systems. (A) The detailed analysis of the components of binding free energies shows that electrostatic interactions dominate the binding (green color), followed by solvation free energies (blue color) and van der Waals (VDW) interactions (red color). (B) The root-mean-square deviation (RMSD) values of the complex backbone atoms of repeated systems with time were similar and fluctuated between 0.37~0.65 nm for all complex systems to reach a plateau after 100 ns MD simulations. (C) The RMSD values of the peptide backbone atoms of repeated systems with time were similar and fluctuated to reach a plateau after 130 ns MD simulations. The RMSD values with time were smoothed by applying 10 snapshots running average.

S3A

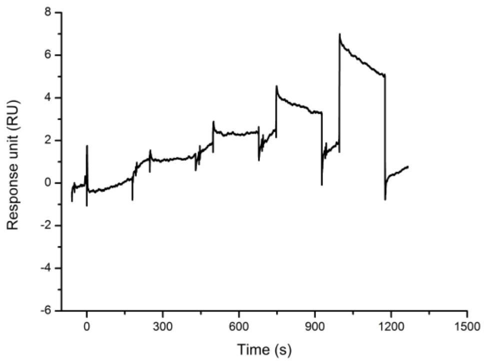

S3B

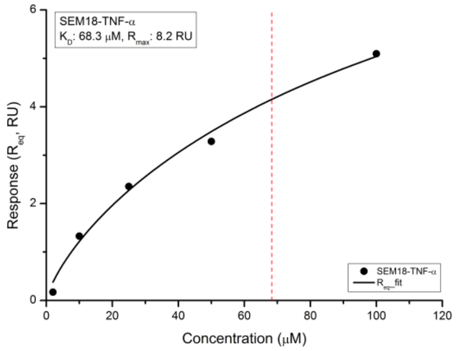

Figure S3: SPR analysis for a truncated peptide SEM18 binding to cytokine TNF-α. (A) SEM18 was injected over TNF-α immobilized on the CM5 sensor chip. As the concentration of SEM18 increased, the measured response for the binding of SEM18 to TNF-α also increased, indicating that binding was concentration dependent. (B) For the steady–state interaction, a binding isotherm was generated to determine the equilibrium K_D and R_{max} for the binding of SEM18 to cytokine TNF-α, which were found to be 68.3 μM and 8.2 RU, respectively.

S4A

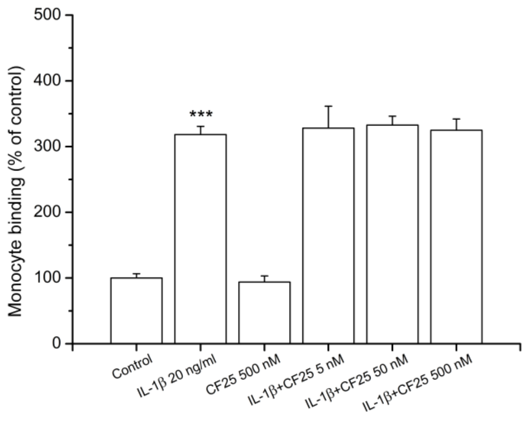
Monocyte binding (% of control)
500
400
300
200
100
0
***
Control
IL-1β 20 ng/ml
CF25 500 nM
IL-1β+CF25 5 nM
IL-1β+CF25 50 nM
IL-1β+CF25 500 nM

S4B

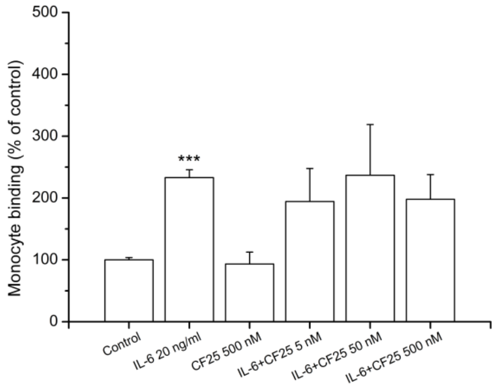
Monocyte binding (% of control)
500
400
300
200
100
0
***
Control
IL-6 20 ng/ml
CF25 500 nM
IL-6+CF25 5 nM
IL-6+CF25 50 nM
IL-6+CF25 500 nM

S4C

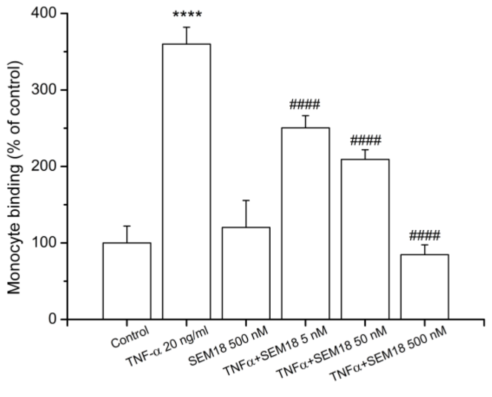
Monocyte binding (% of control)
400
300
200
100
0
****
####
####
####
Control
TNF-α 20 ng/ml
SEM18 500 nM
TNFα+SEM18 5 nM
TNFα+SEM18 50 nM
TNFα+SEM18 500 nM

S4D

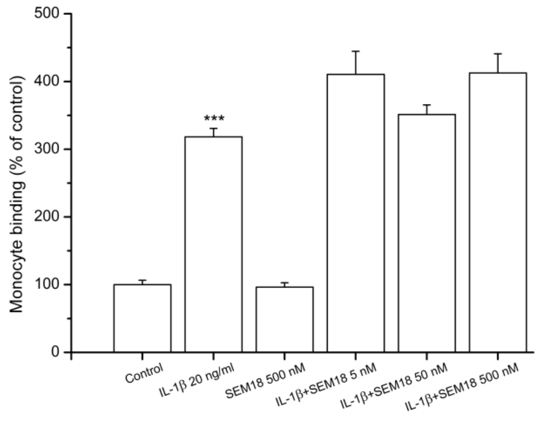

S4E

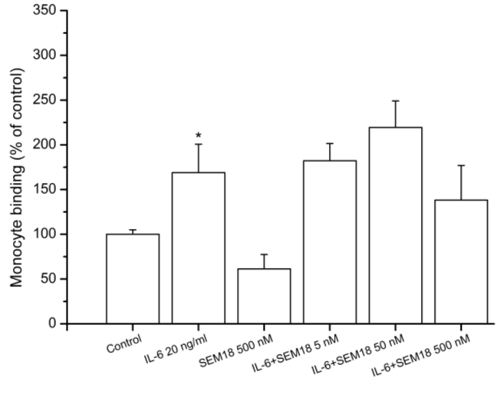

Figure S4: Comparison of various concentrations of peptides CF25 and SEM18 to inhibit cytokines induced monocyte adhesion to HMEC-1. HMEC-1 was pretreated with various concentrations of the peptide CF25 for one hour, and then stimulated with 20 ng/ml (A) IL-1β (B) IL-6 for 18 hours. HMEC-1 cells were pretreated with various concentrations of SEM18 for 1 hour and were then stimulated with 20 ng/mL (C) TNF-α, (D) IL-1β, or (E) IL-6 for 18 hours. Adhesion of fluorescent THP-1 cells was photographed by fluorescent microscopy and calculated. “Control” means that only the culture medium (without peptides) is incubated with cells. Values are mean ± SD from three independent experiments. *$P < 0.05$, **$P < 0.01$, ***$P < 0.001$, and ****$P < 0.0001$

as compared with control; #P< 0.05, ##P < 0.01, ###P < 0.001, and ####P < 0.0001 as compared with cells stimulated with cytokines in the absence of the peptides.

S5

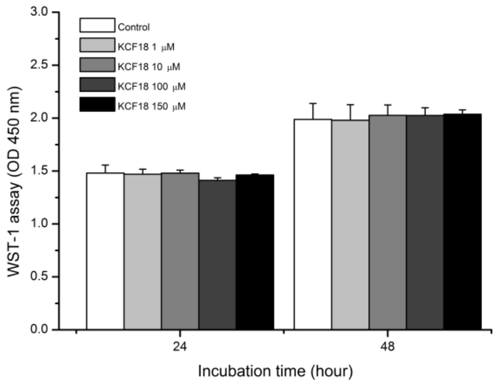

Figure S5: Peptide KCF does not induce the cytotoxic effect on endothelial cells. HMEC-1 was treated with various concentrations of peptide KCF in 96-well plate. After 24 and 48 hours of incubation, cell viability was evaluated using the colorimetric WST-1 assay. Data are the mean ± SD of triplicate determinations. “Control” means that only the culture medium (without peptides) is incubated with cells.

S6A

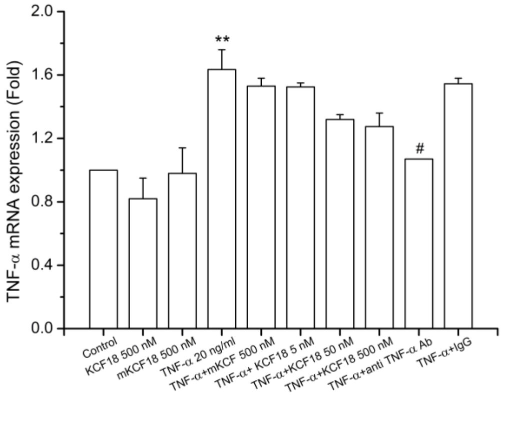

S6B

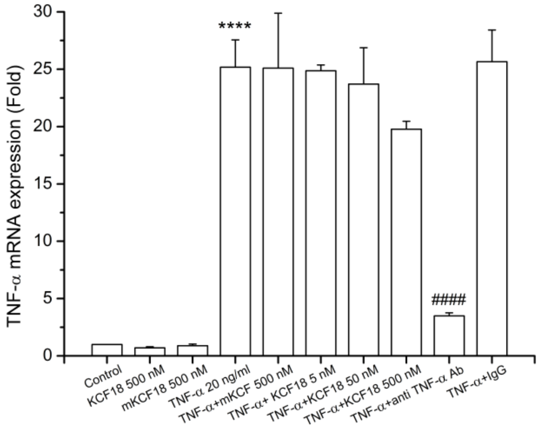

Figure S6: Peptide KCF18 and TNF-α antagonist antibody affect the mRNA expressions of cytokine-induced TNF-α in HMEC-1 and THP-1 cells. TNF-α mRNA levels induced by TNF-α in (A) THP-1 and (B) HMEC-1 were determined using qPCR assays, as described in Materials and Methods. GAPDH cDNA was used as an internal control. Values are mean ± SD of mRNA levels relative to those for GAPDH from three independent experiments. *P < 0.05, **P < 0.01, ***P< 0.001, and ****P< 0.0001 as compared with control; #P< 0.05, ##P < 0.01, ###P < 0.001, and ####P < 0.0001 as compared with cells stimulated with cytokines in the absence of the peptides.

S7A

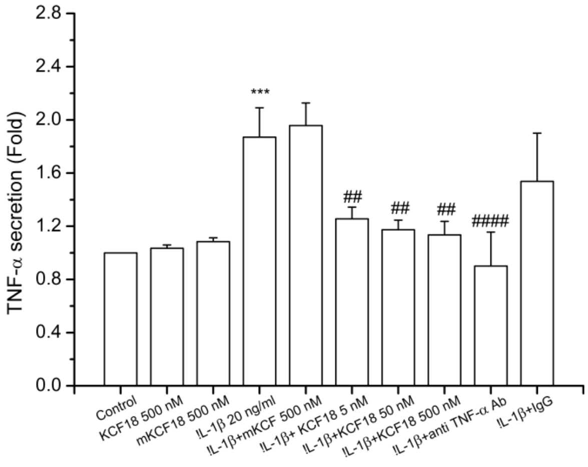

S7B

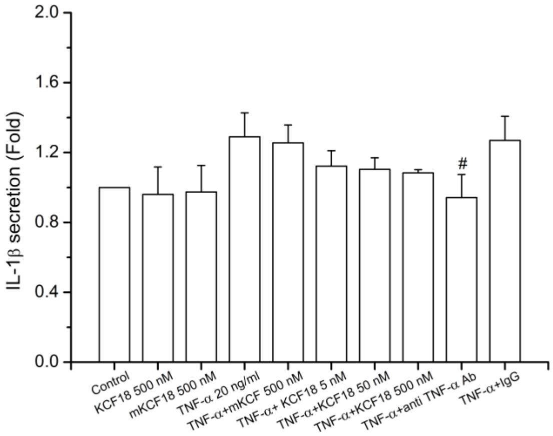

Figure S7: Peptide KCF18 and TNF-α antagonist antibody affect the protein expression of cytokines-induced TNF-α and IL-1β in THP-1 cells. TNF-α and IL-1β secretion levels induced by (A) IL-1β and (B) TNF-α in THP-1 were determined using ELISA assays. Total cell lysate concentrations were used as an internal control. Values are mean ± SD of cytokine levels relative to those for cell lysate concentrations from three independent experiments. *P < 0.05, **P < 0.01, ***P< 0.001, and ****P< 0.0001 as compared with control; #P< 0.05, ##P < 0.01, ###P < 0.001, and ####P < 0.0001 as compared with cells stimulated with cytokines in the absence of the peptides.

S8A

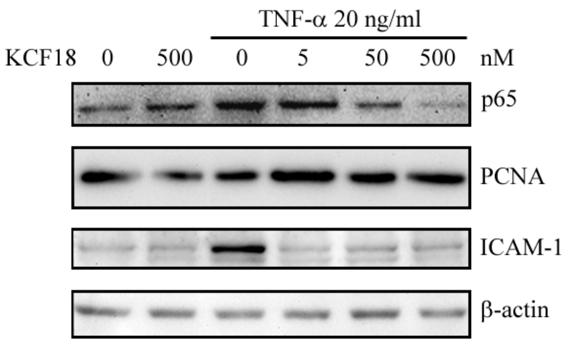
TNF-α 20 ng/ml
KCF18 0 500 0 5 50 500 nM
p65
PCNA
ICAM-1
β-actin

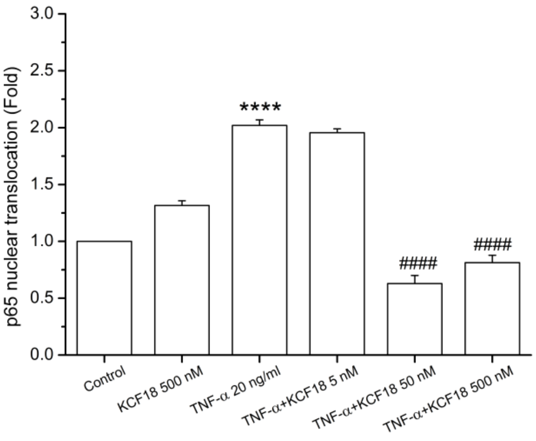
p65 nuclear translocation (Fold)
3.0
2.5
2.0
1.5
1.0
0.5
0.0
****
####
####
Control
KCF18 500 nM
TNF-α 20 ng/ml
TNF-α+KCF18 5 nM
TNF-α+KCF18 50 nM
TNF-α+KCF18 500 nM

S8B

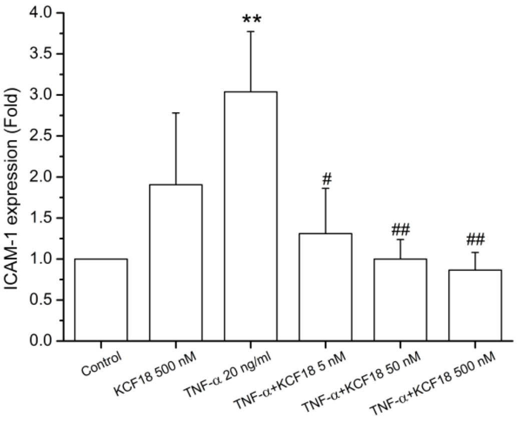
ICAM-1 expression (Fold)
4.0
3.5
3.0
2.5
2.0
1.5
1.0
0.5
0.0
**
#
##
##
Control
KCF18 500 nM
TNF-α 20 ng/ml
TNF-α+KCF18 5 nM
TNF-α+KCF18 50 nM
TNF-α+KCF18 500 nM

Figure S8: Peptide KCF18 reduces the TNF-a-induced p65 nuclear translocation and ICAM-1 expression in HMEC-1 cells. (A) p65 nuclear translocation and (B) ICAM-1 expression in HMEC-1 cells were determined using Western blotting. PCNA as an internal control for p65 nuclear translocation and b-actin as an internal control for ICAM-1 expression. Data presented here are representative image of two independent experiments. The significance levels *P < 0.05, **P < 0.01, ***P< 0.001, and ****P< 0.0001 as compared with control; #P< 0.05, ##P < 0.01, ###P < 0.001, and ####P < 0.0001 as compared with cells stimulated with cytokines in the absence of the peptides.

S9

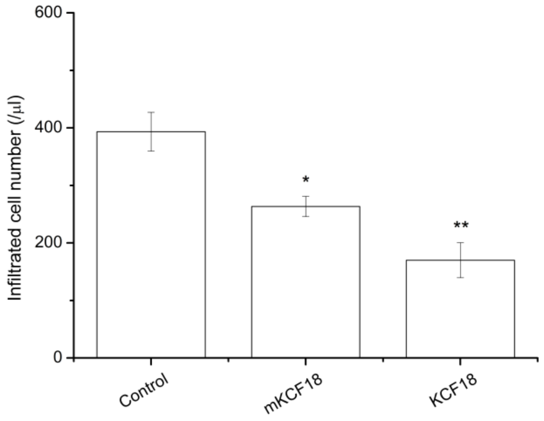

Figure S9: KCF18 alleviates peritonitis compared with mutant peptide mKCF18.

Cell counts from peritoneal fluid 24 hours after intraperitoneal injection of thioglycollate in mice are shown. Comparisons of KCF18 with mKCF18 peptide. Values are mean ± SEM (n = 3 for each group). *P < 0.05 and **P < 0.01 as compared with control.

Here are the loading sequences of the original images. Data presented here are representative image of two independent experiments.

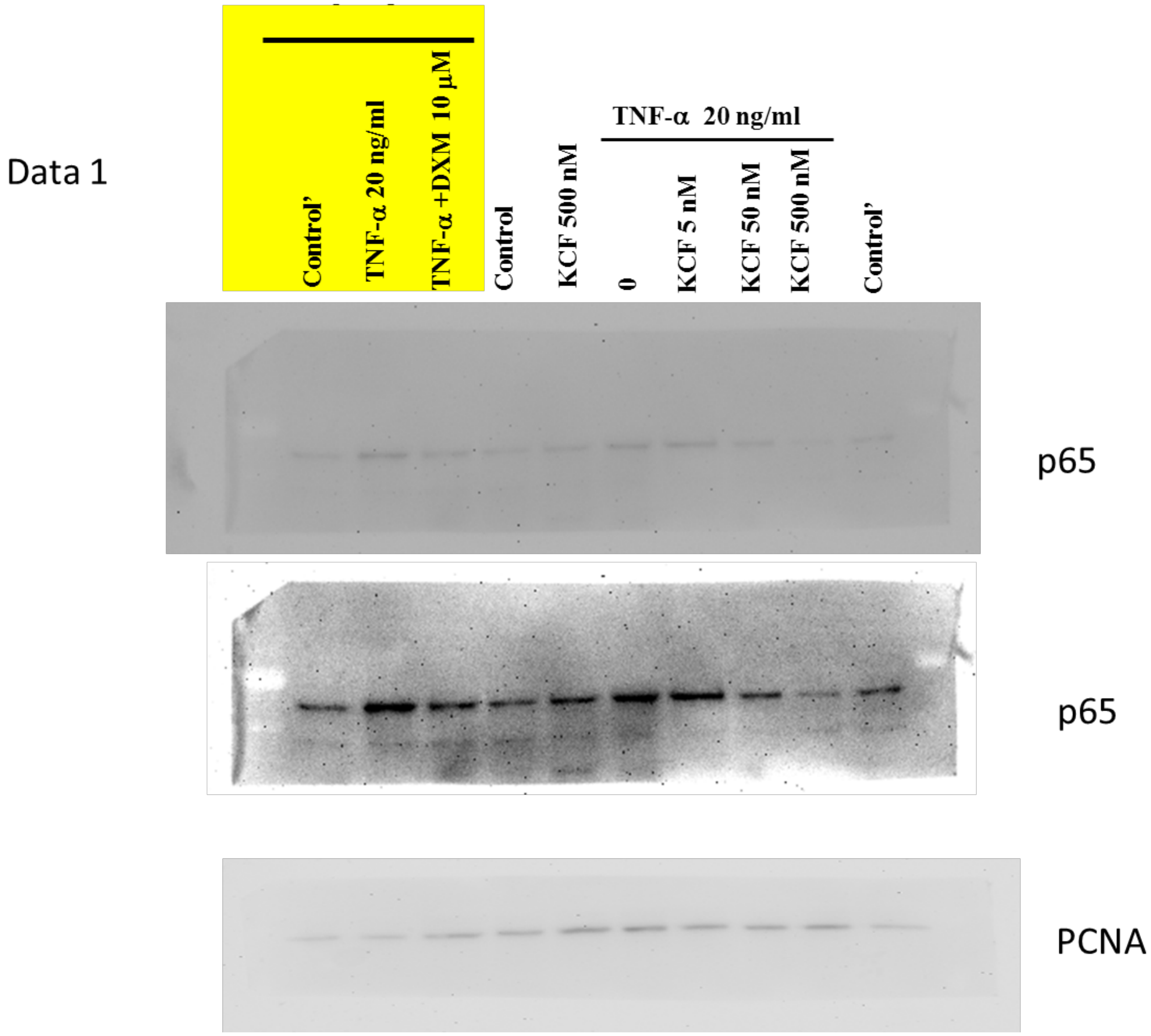

Data 2

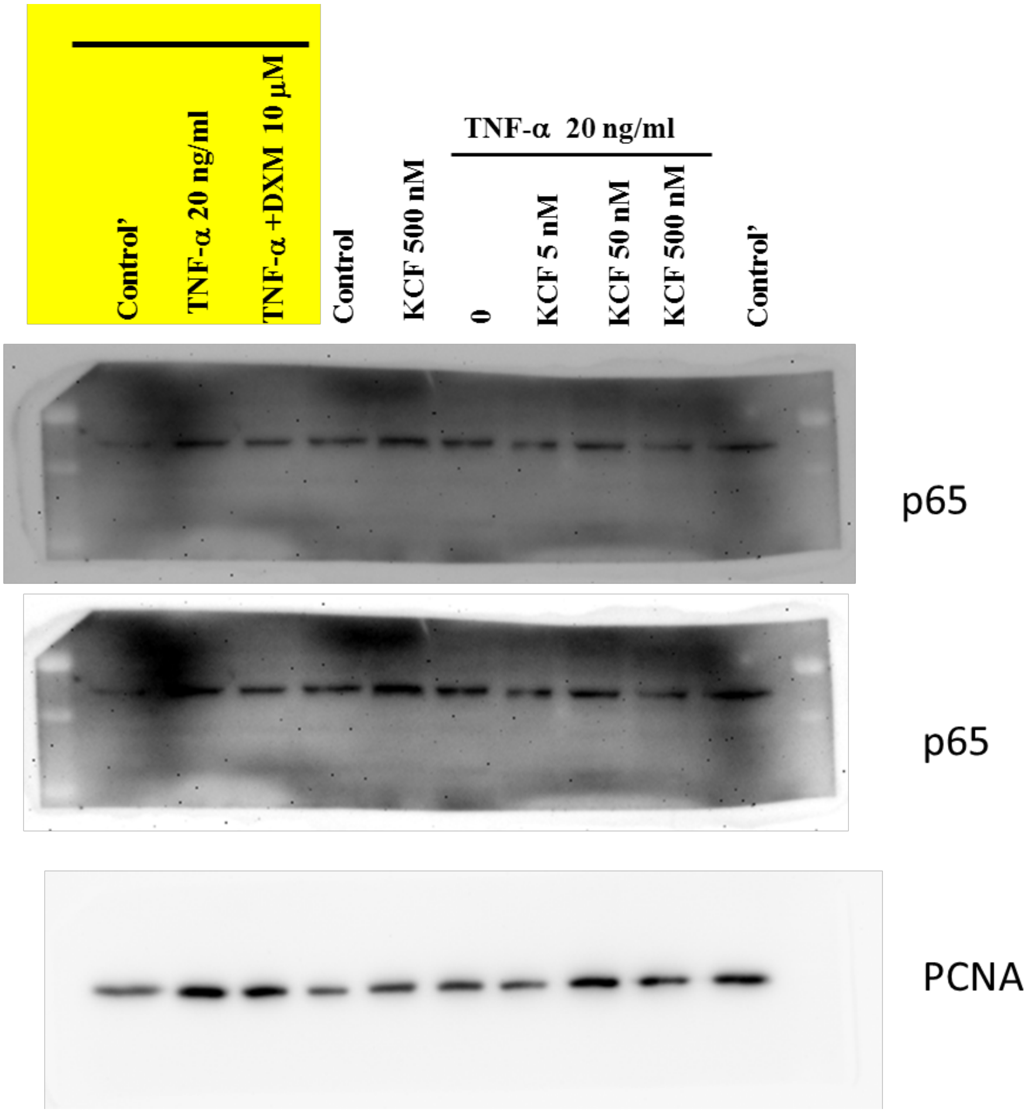
Control'
TNF-α 20 ng/ml
TNF-α +DXM 10 μM
Control
KCF 500 nM
TNF-α 20 ng/ml
0
KCF 5 nM
KCF 50 nM
KCF 500 nM
Control'
p65
p65
PCNA

Data 1

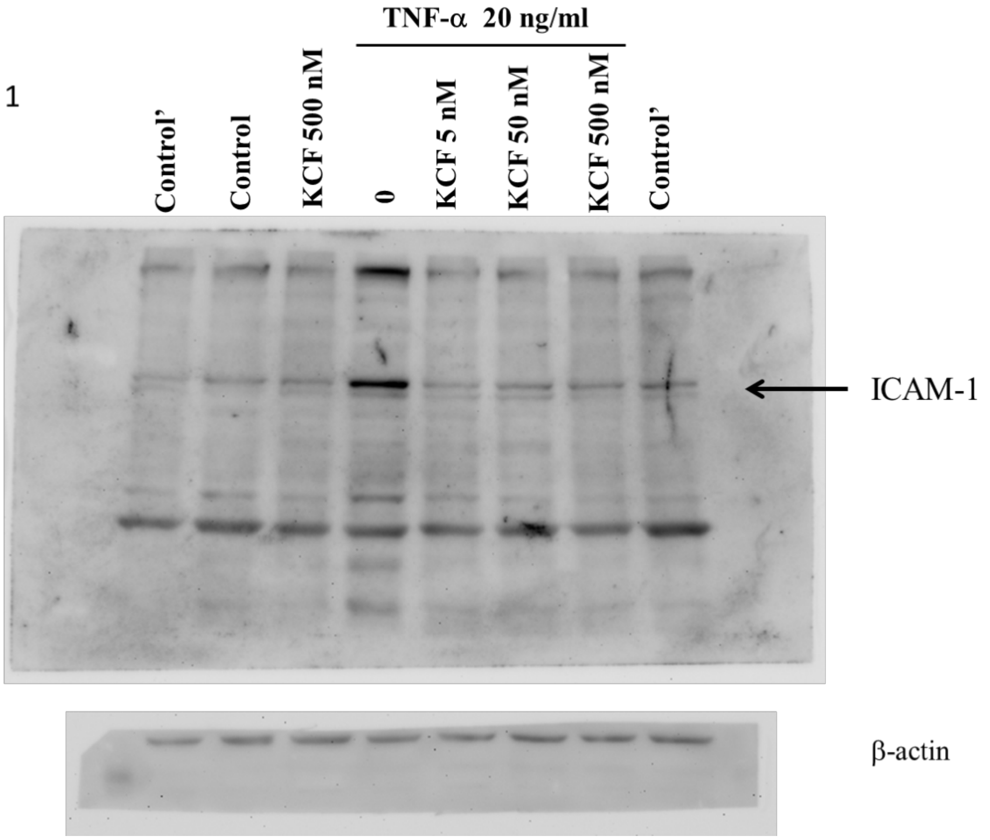
TNF-α 20 ng/ml
Control'
Control
KCF 500 nM
0
KCF 5 nM
KCF 50 nM
KCF 500 nM
Control'
ICAM-1
β-actin

Data 2

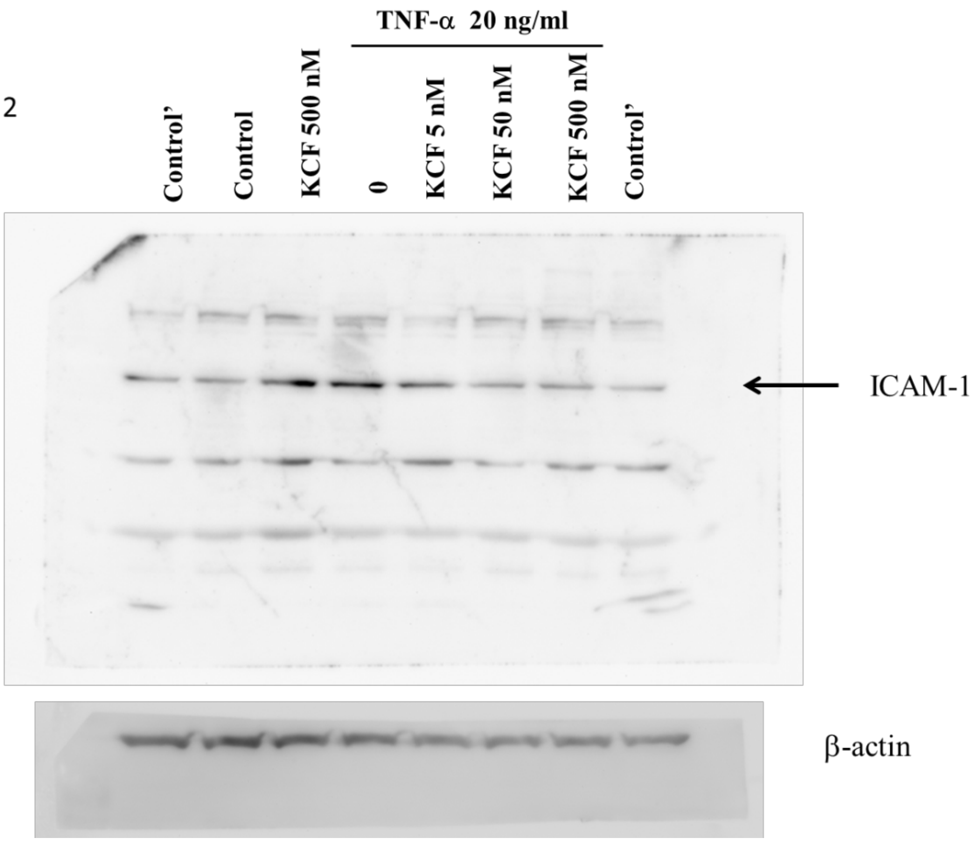
TNF-α 20 ng/ml
Control'
Control
KCF 500 nM
0
KCF 5 nM
KCF 50 nM
KCF 500 nM
Control'
ICAM-1
β-actin

Table S1. The computational alanine mutation calculations

(kJ/mol)	ΔG	ΔΔG
**KCF18-TNF-α**	-583.6 ± 3.6	0 ± 0
p_K1A	-616.9 ± 3.3	-33.3 ± 4.9
p_R3A	-557.9 ± 3.1	25.7 ± 4.8
p_K4A	-542.4 ± 2.9	41.2 ± 4.6
p_K8A	-568.5 ± 2.8	15.1 ± 4.6
p_K10A	-603.4 ± 3.1	-19.8 ± 4.8
**KCF18- IL1β**	-394.9 ± 3.8	0 ± 0
p_K1A	-345.1 ± 3.3	49.8 ± 5.0
p_R3A	-315.2 ± 3.5	79.7 ± 5.2
p_K4A	-429.9 ± 3.8	-35.0 ± 5.4
p_K8A	-431.0 ± 3.9	-36.1 ± 5.4
p_K10A	-271.3 ± 4.7	123.6 ± 6.0
**KCF18- IL-6**	-526.6 ± 5.3	0 ± 0
p_K1A	-551.5 ± 5.0	-24.9 ± 7.3
p_R3A	-508.9 ± 5.2	17.7 ± 7.4
p_K4A	-603.6 ± 5.3	-77.0 ± 7.4
p_K8A	-470.3 ± 5.3	56.3 ± 7.5
p_K10A	-548.4 ± 5.5	-21.8 ± 7.6
